# A comparative review on Ayam Cemani chicken — A comparison with the most common chicken species in terms of nutritional values, LCA, price and consumer acceptance

**DOI:** 10.1007/s11250-024-03980-6

**Published:** 2024-05-11

**Authors:** Shahida Anusha Siddiqui, Valeria Toppi, Layyinatus Syiffah

**Affiliations:** 1 Technical University of Munich, Campus Straubing for Biotechnology and Sustainability, Essigberg 3, 94315 Straubing, Germany; 2 German Institute of Food Technologies (DIL e.V.), Prof.-von-Klitzing Str. 7, 49610 Quakenbrück, Germany; 3 Department of Veterinary Medicine, University of Perugia, Via S. Costanzo 4, 06126 Perugia, Italy; 4 Nutrition Science Department, Faculty of Medicine, Diponegoro University, Semarang, 50275 Indonesia

**Keywords:** Ayam Cemani, Black birds, Rare breed, Markets, Black meat

## Abstract

Chickens are definitely among the most prevalent and broadly distributed domestic species. Among these, Ayam Cemani, also known as black chicken, is a rare Indonesian chicken breed originating from the island of Java. The main characteristic of this breed is that the body, both internally and externally, is entirely black. This is due to a condition named fibro melanosis, in which there is an over accumulation of melanin pigment in body tissues. In addition to this, Ayam Cemani meat results to be also higher in protein content and lower in fat. Moreover, Ayam Cemani meat is also known to have antioxidant and glucose-binding capacities. These properties make it very desirable within the market and consequently very expensive. Their meat is also used traditionally by tribal healers in the treatment of some chronic illnesses. In general, compared to other chicken species, the Ayam Cemani showed an higher genetic resistance to some infectious diseases commonly affecting poultry species. As regard the breeding, Ayam Cemani is a unique breed which may only be raised in specific locations, characterized to be a slowly growing breed with a lower body weight in comparison to the other poultry breeds. Nowadays, due to an improvement in the management, the nutrition and diseases control, it is possible to enhance their productivity. To date, there are not many studies in the literature on the specific breed of Ayam Cemani. For this reason, this review aims to provide a comprehensive overview of all the knowledge of the Ayam Cemani breed, the nutritional composition of the meat and consumer acceptance.

**Graphical abstract:**

Overview of Ayam Cemani origin and uses

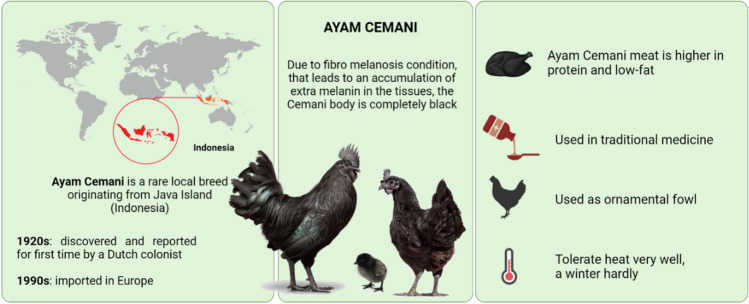

## Introduction

Chicken is definitely the most numerous and widely distributed among the domesticated livestock species (Sulundari et al. 2008; Lawal and Hanotte 2021a). Unlike other domestic animal species, chickens are by far accepted without or with few taboos in the world’s different religions, societies and cultures (Lawal and Hanotte 2021a).

Ayam Cemani (black chicken) is a rare local breed, originated from the Kedu Village, Temanggung city, Java Island (Indonesia). The name of this breed comes from the union of Indonesian words, “Ayam” meaning “chicken” and “Cemani” meaning “entirely black”. The origin of the Ayam Cemani chicken breed is still unclear (Dharmayanthi et al. 2022). The first evidence of this breed is reported by a Dutch colonist living in Java during the 1920s. Then, in the 1990s the Ayam Cemani chicken breed figured in Europe and thus in the United States. In the past, due to rarity, the price to buy these exotic birds was $2000. Nowadays, price has come down a lot and it is possible to buy an Ayam Cemani chick for under $50. However it is not easy to find hatcheries that supply them, and most of times chicks are flawed as they are not pure or not completely black.

To date, it is universally recognized that all populations of domesticated chicken descend from a single ancestor, called the Red Jungle Fowl (*Gallus gallus*, RJF), originating from Southeast Asia. Domestic (also known as indigenous) chickens present in throughout the archipelago of Indonesia are characterized by their great diversity and different morphological features. Indonesia is a country of unprecedented wealth in genetic resources, especially in those concerning the native chickens (Hidayat and Asmarasari 2015). In fact, in Indonesia there are now identified 31 breeds of indigenous chickens (Table 2). Of these, 11 chicken breeds are mentioned as chicken with an higher production of eggs. Twelve are part of the ornamental chicken breeds, according to their voice. Moreover, they are also known as fighting cocks. Chicken breeds classified as broiler are four. Finally, there are a list of 9 breeds of chicken, which their superiority has not been discovered yet (Hidayat and Asmarasari 2015).

The most representative feature of Ayam Cemani chicken is that they are entirely black, both externally and internally. This condition is due to the hyper melanic pigmentation affecting the entire body, including the plumage, comb, shank, tongue and eye. In addition, it is possible to find this hyperpigmentation in the most of the inner body, like in the muscles, intestines, bones, peritoneum and also trachea. This unique characteristic is due to the phenotype known as Fibromelanosis (*Fm*). Other than Silkie breed, the Ayam Cemani is known to be the representative chicken breed carrying the *Fm* mutation Dharmayanthi et al., 2017. Accordingly to the fact that it is entirely black from the skin to the bond (Prakash et al. 2023). All over the world, Ayam Cemani is the only animal listed as black food.

Since the beginning, the relationship between Indonesian people and native chickens has always been very close. As far back as ancient times native chickens were largely employed as integrant part of culture and customs of Indonesian inhabitants. Indeed, for Indonesian small holder farmers, as is the case with other developing nations, indigenous chickens represent one of the few chances to save, invest and secure against possible risks. The Indonesian native chickens have been classified according to their potential use in: meat and egg production, singing chickens, fancy chickens, chickens used for traditional medicine and also fighting cocks. According to a more recent division, the indigenous Indonesian chicken can be classified into four functional groups as singing chicken, used in traditional ceremonies, fancy and fighting cock and meat and egg producers (Hidayat and Asmarasari 2015).

Two different theories have been proposed to explain the origin of Indonesian indigenous chicken. In the first one, defined as “monophyletic origin” it has been reported that native chickens originated from one ancestor. Instead, the second one theory, called “polyphyletic origin”, which explains explain how native chickens derive from several common ancestors (Hidayat and Asmarasari 2015). In a work conducted by Sulandari et al. (2009), it is reported that Indonesian native chickens are the result of a domestication process of the Red Jungle Fowl (RJF). On the other hand, Muladno and Thieme (2009) considered the origin of indigenous chickens as a result from the domestication process of four different wild chicken species, such as green wild chicken (*Gallus various*), Red Wild Chicken (*Gallus gallus*), Indian Grey Wild Chicken (*Gallus soneratti*) and at least Ceylon Orange Wild Chicken (*Gallus lavayetti*). Also Sulandari et al. (2009) stated that the one of the most important centre of chicken domestication all around the world is Indonesia.

**Fig. 1:**
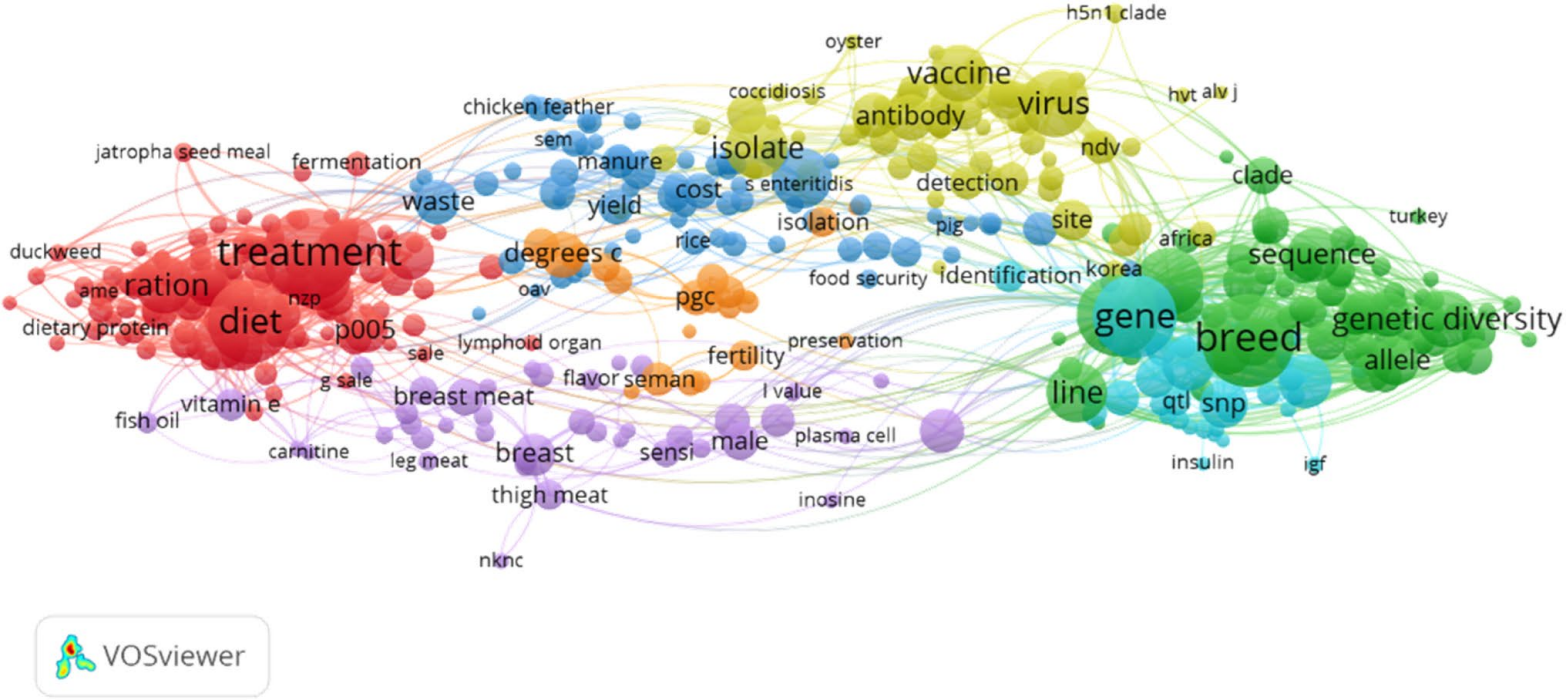
Scientific research bibliometric map on Ayam Cemani chicken

In Indonesia, the price of native chicken meat is higher that the broiler one. This is due to the fact that native chicken product has a good brand in Indonesian market. In fact, the meat of indigenous chicken has a specific texture and taste that makes it the preferred choice by the majority of Indonesians. In addition, also the native chicken eggs are more expensive than the layer eggs. This is due to the fact that, beside to the normal consume, the native chicken eggs are also used as part of the very popular traditional herbal medicine known as “Jamu”. However, among the main obstacles to development of native chicken there is the low production performance. Native chickens are characterized by low growth rate, resulting in small carcass percentage, low body weight, risks of high mortality due to the presence of Newcastle disease, low body and egg size, low hen day and hen house and low egg production. In response to all these factors, and with the aim to improve the quality of native chicken, many researchers have stated that there is the necessity to improve the breeding, the feeding and the management to allow an increase in the native chicken productivity. A possibility is that an improvement in genetic quality through cross breeding or selection process can enhance the production of native chicken. Moreover, also an improvement in the feeding system is responsible for an increase in the production of meat and egg by indigenous chicken (Fig. 1).

## Overview of chicken species worldwide and focus on Ayam Cemani breed

### The characteristics of the world’s main chicken breeds and their classification

**Fig. 2:**
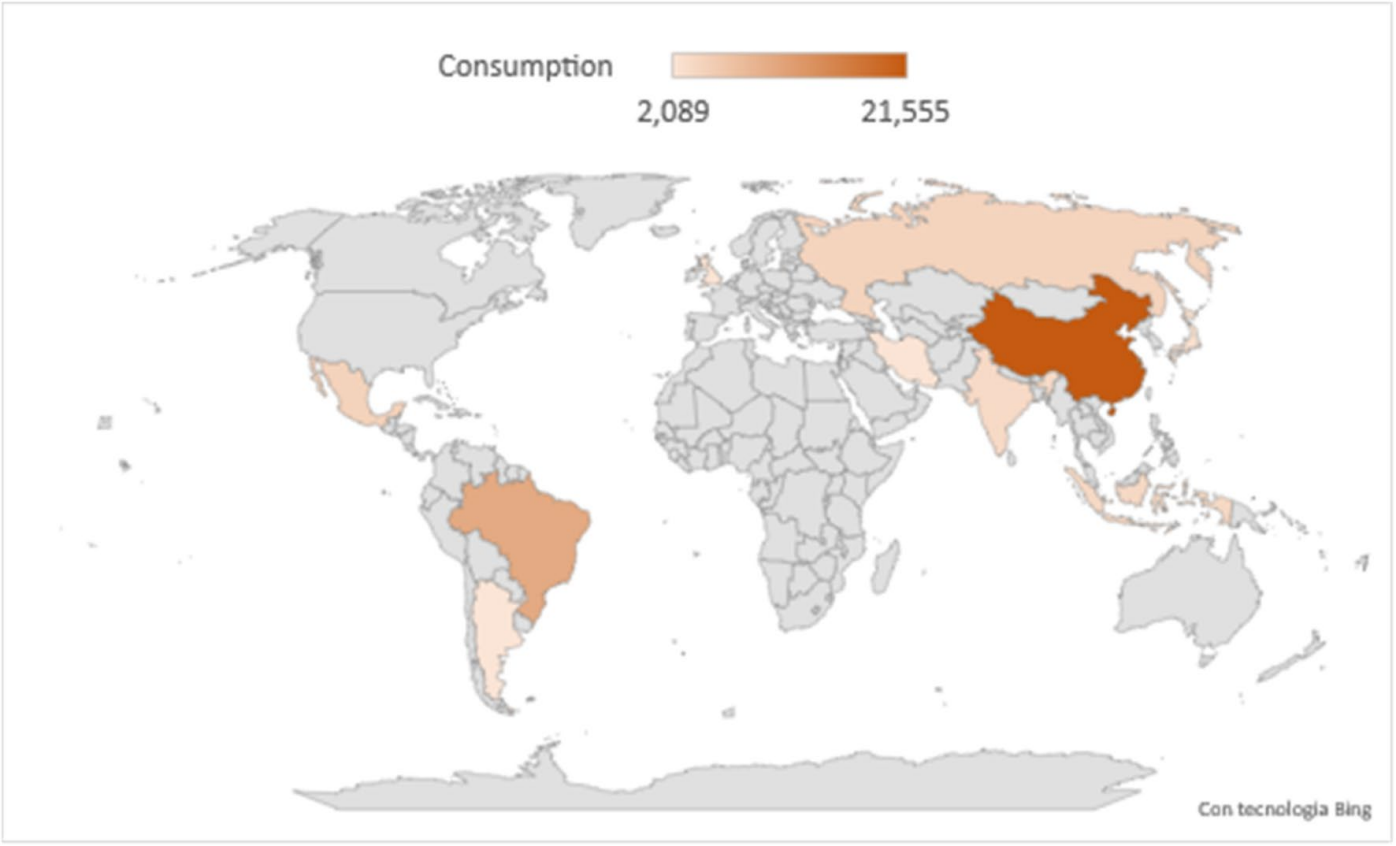
The figure highlights the states with the highest consumption of chicken meat. Orange intensity indicates the number of chicken meat (in tonnes) consumed per state

Many types of chicken are raised throughout the world (Fig. 2). In fact to date, more than 350 combinations of physical features for chicken species are known. In order to make a classification and a proper identification, all chicken species are sorted by classes, breeds, varieties and at least strains. In Table 1, different types of chickens are listed based on their classes. The classification reported below is based on Skinner and Hady (2018). Briefly, a class is a group of breed having in common same characteristics, such as the American, the Asiatic, the English, the Mediterranean and the Continental class whereas breed indicates a group with a specific set of physical characteristics such as body type or shape, colour of the skin, number of toes, feathered/not feathered shanks. As shown in Table 1, there are five breeds in the American class, three in the Asiatic, four in the English, four in the Mediterranean, six in the Continental one and five breeds in the all-other standard breed category.

**Table 1:** Classification of chicken species worldwide

Class	Breed	Variety	Standard Weights	Skin colour	Eggshell colour	Use	Specific use	Origin	Special characteristics	References
American	Jersey Giant	Black, Blue, White	Cock: 13 pounds Hen: 10 pounds Cockerel: 11 pounds Pullet: 8 pounds	Yellow	Brown	Dual purpose	Used mainly for meat and capon production	New Jersey	Largest breed in the American Class. Rugged, with angular shape, single comb and black shanks in the White variety. Not the best choice for incubating/brooding. Meat yield is disappointing until 6 months or older	(Skinner and Hady 2018)
	New Hampshire red	-	Cock: 8½ pounds Hen: 6½ pounds Cockerel: 7½ pounds Pullet: 5½ pounds	Yellow	Brown	Dual purpose	Meat and eggs production	Massachusetts and New Hampshire	Deep, broad body, grow feathers. Good for brooding. The color is a medium to light red. The comb is single and medium to large in size. Fair egg-laying ability. Competitive and aggressive	(Skinner and Hady 2018)
	Plymouth Rock	Barred, Blue, Buff, Columbian, Partridge, Silver Penciled, White	Cock: 9½ pounds Hen: 7½ pounds Cockerel: 8 pounds Pullet: 6 pounds	Yellow	Brown	Dual purpose	Used as principal stock for American broiler production; Used also as pets	United States	Good general farm chickens. Docile and good for brooding. Long, broad back, moderately deep, full breast, single comb of moderate size. Not extremely aggressive, tame quite easily. Wide and straight back. Common faults: shallow breast, high tails, narrow bodies and small size	(Skinner and Hady 2018)
	Rhode Island Red	Rose Comb, Single Comb	Cock: 8½ pounds Hen: 6½ pounds Cockerel: 7½ pounds Pullet: 5½ pounds	Yellow	Brown	Dual purpose	Used mainly as a layer breed; Also used in the creation of many modern hybrid breeds	New England states of Massachussets	Best egg-layers. Some males quite aggressive. Rectangular, relatively long bodies, typically dark red in color	(Skinner and Hady 2018)
	Wyandotte	Black, Blue, Buff, Columbian, Golden Laced, Partridge, Silver Laced, Silver Penciled, White	Cock: 8½ pounds Hen: 6½ pounds Cockerel: 7½ pounds Pullet: 5½ pounds	Yellow	Brown	Dual purpose	Used for meat and eggs production; Also used as show bird, particularly in Germany	United States	Good, medium-weight fowl. Their rose combs do not freeze easily. Hens are good mothers. Common faults: narrow backs, undersized individuals, relatively poor hatches	(Skinner and Hady 2018)
Asiatic	Brahman	Buff, Dark, Light	Cock: 12 pounds Hen: 9½ pounds Cockerel: 10 pounds Pullet: 8 pounds	Yellow	Brown	Dual purpose	Used mainly for meat and capon production	China	Large size and gentle nature, intricated color pattern. Good for brooding and good mothers. Able to resist to cold temperatures with their small comb and wattles, profuse feathering and well-feathered shanks and toes. Slow rate to growth and reach maturity	(Skinner and Hady 2018)
	Cochin	Barred, Black, Blue, Brown, Buff, Golden Laced, Partridge, Silver Laced, White	Cock: 11 pounds Hen: 8½ pounds Cockerel: 9 pounds Pullet: 7 pounds	Yellow	Brown	Ornamental	Used mainly for exhibition; Hens are often used as foster mothers. Rarely used for meat production	China	Need to confine them during wet days and with muddy yards due to their profuse leg and foot feathering. Persistent broodiness and intense layers for short period. Artificial insemination required to obtain good rate of fertility	(Skinner and Hady 2018)
	Langshan	Black, Blue, White	Cock: 9½ pounds Hen: 7½ pounds Cockerel: 8 pounds Pullet: 6½ pounds	White	Brown	Dual purpose	Used also as ornamental fowl	China	Very tall, long legs and tails carried at high angle. Active and quick. Good general breed and good mothers	(Skinner and Hady 2018)
English	Australorp	Black	Cock: 8½ pounds Hen: 6½ pounds Cockerel: 7½ pounds Pullet: 5½ pounds	White	Brown	Dual purpose	Used mainly for eggs production	Australia	Intense beetle-green sheen on the black birds, dark eyes, deep bodies, very active. Good eggs producers	(Skinner and Hady 2018)
	Cornish	Buff, Dark, White, White Laced Red	Cock: 10½ pounds Hen: 8 pounds Cockerel: 8½ pounds Pullet: 6½ pounds	Yellow	Light Brown	Dual purpose	Used mainly for eat production in broiler industry worldwide	England	Broad and well-muscled body. Legs with large diameter and widely spaced. Deep-set eyes, cruel expression. Need protection during cold temperatures. Poor fertility. Movers, needing of space to exercise their muscles. Very protective mothers but too active to be good brood hens	(Skinner and Hady 2018)
	Orpington	Black, Blue, Buff, White	Cock: 10 pounds Hen: 8 pounds Cockerel: 8½ pounds Pullet: 7 pounds	White	Brown	Dual purpose	Used as ornamental fowl and pet	England	Heavily but loosely feathered, massive appearance. Resistant to cold temperatures. Hens show broodiness and are generally good mothers. Chicks not aggressive	(Skinner and Hady 2018)
	Sussex	Light, Red, Speckled	Cock: 9 pounds Hen: 7 pounds Cockerel: 7½ pounds Pullet: 6 pounds	White	Brown	Dual purpose	Used also as pet	England	Alert, attractive and good foragers. Rectangular bodies. Go broody and good mothers	(Skinner and Hady 2018)
Mediterranean	Andalusian	Blue	Cock: 7 pounds Hen: 5½ pounds Cockerel: 6 pounds Pullet: 4½ pounds	White	White	Ornamental fowl	Used for eggs production	Spain	Small, active, closely feathered birds, noisy and rarely go broody. Unstable blue color. Not largely bred	(Skinner and Hady 2018)
	Leghorn	Rose Comb: Black, Buff, Dark, Brown, Light Brown, Silver White; Single Comb: Black, Black Tailed Red, Buff, Columbian, Dark Brown, Golden, Light Brown, Red, Silver, White	Cock: 6 pounds Hen: 4½ pounds Cockerel: 5 pounds Pullet: 4 pounds	Yellow	White	Eggs production	Much used to create highly productive egg-laying hybrids for commercial and industrial purpose	Italy	Small, spritely, noisy bird. Good foragers. Able to considerable flight. Most numerous breed in United States. Rarely go broody	(Skinner and Hady 2018)
	Minorca	Rose Comb: Black, White; Single Comb: Black, Buff, White	Cock: 9 pounds Hen: 7½ pounds Cockerel: 7½ pounds Pullet: 5½ pounds	White	White	Ornamental fowl	Used mainly for showing	Spain	Largest mediterranean breed, long angular birds. Long tails and large wide feathers. Relatively large combs and wattles. Rather poor meat, rarely go broody, very alert and fairly good foragers	(Skinner and Hady 2018)
	White Faced Black Spanish	-	Cock: 8 pounds Hen: 6½ pounds Cockerel: 6½ pounds Pullet: 5½ pounds	White	Chalky white	Ornamental fowl	Eggs production	Spain	Large area of snow-white skin surrounding the face and wattles. Non-broody. Active and noisy	(Skinner and Hady 2018)
Continental	Hamburg	Black, Golden Penciled, Golden Spangled, Silver Penciled, Silver Spangled, White	Cock: 5 pounds Hen: 4 pounds Cockerel: 4 pounds Pullet: 3½ pounds	White	White	Ornamental fowl	Eggs production	Holland	Active and flighty birds. Trim and stylish with delicate features and wild in nature. Forage well and flight for long distances. Good egg producers. Eggs very small	(Skinner and Hady 2018)
	Welsummer	-	Cock: 7 pounds Hen: 6 pounds Cockerel: 6 pounds Pullet: 5 pounds	Yellow	Dark brown	Dual purpose	Mainly used for eggs production	Holland	Distinctive marking and colouring. Big-boodied with a good number of eggs produced. Non sitting fowl	(Skinner and Hady 2018)
	Polish	Bearded: Buff Laced, Golden, Silver, White; Non-Bearded: Black Crested White, Buff Laced, Golden, Silver, White, White Crested Black, White Crested Blue	Cock: 6 pounds Hen: 4½ pounds Cockerel: 5 pounds Pullet: 4 pounds	White	White	Ornamental fowl	Originally breed for eggs production, now used mainly for showing	Eastern Europe	Unusual breed. Crest (some beard and muffs). Small, tightly feathered birds and fairly active. Need of plenty of space. No adapted to cold	(Skinner and Hady 2018)
	Faverole	Salmon, White	Cock: 8 pounds Hen: 6½ pounds Cockerel: 7 pounds Pullet: 5½ pounds	White	White	Dual purpose	Used mainly for showing	France	Beards and muffs with single comb and feathered legs and feets. Medium-size breed and fairly loosely feathered. Fifth toe on each feet	(Skinner and Hady 2018)
	Houdan	Mottled, White	Cock: 8 pounds Hen: 5½ pounds Cockerel: 7 pounds Pullet: 5½ pounds	White	White	Dual purpose	Used mainly as ornamental fowl	France	Crest, beard and muffs and five toes each feet. Rectangular bodies. Require lot of space, feed and space containers. Baby Houdans often walk with skipping gait	(Skinner and Hady 2018)
	Maran	Black Copper, Wheaten, White	Cock: 8 pounds Hen: 6½ pounds Cockerel: 7 pounds Pullet: 5½ pounds	White	Very dark reddish brown	Dual purpose	Used mainly for meat and eggs production	France	Medium size	(Skinner and Hady 2018)
All other standard breeds (Miscellaneous)	Old English Game	Black, Black Breasted Red, Blue Breasted Red, Blue Golden Duckwing, Blue Silver Duckwing, Lemon Blue, Red Pyle, Self Blue, Silver Duckwing, Spangled, White	Cock: 5 pounds Hen: 4 pounds Cockerel: 4 pounds Pullet: 3½ pounds	White	White or light tint	Ornamental fowl	Mainly used for showing	England	Small, tightly feathered bird. Very hardy, active and noisy. Broodiness but they are aggressive and defensive. Capable to considerable flight and revert to feral state in some areas	(Skinner and Hady 2018) (Skinner and Hady 2018)
	Modern Game	Birchen, Black, Black Breasted, Brown Red, Golden Duckwing, Red Pyle, Silver Duckwing, Wheaten, White	Cock: 6 pounds Hen: 4½ pounds Cockerel: 5 pounds Pullet: 4 pounds	White	White or light tint	Ornamental fowl	Mainly used for showing or as a pet	Great Britain	Tightly feathered bird with long neck and legs. No adapted to cold temperatures and needing plenty of exercise to maintain muscle tone	(Skinner and Hady 2018)
	Malay	Black, Black Breasted Red, Red Pyle, Spangled, Wheaten, White	Cock: 9 pounds Hen: 7 pounds Cockerel: 7 pounds Pullet: 5 pounds	Yellow	Brown	Ornamental fowl	Mainly used for showing	Asia	Very tall, bold and cruels. Closely feathered. Vigor and long life. Most hens go broody but not best choice because of their long legs	(Skinner and Hady 2018)
	Sumatra	Black, Blue	Cock:5 pounds Hen: 4 pounds Cockerel: 4 pounds Pullet: 3½ pounds	Yellow	White or light tint	Ornamental fowl	Strictly ornamental or pet	Island of Sumatra	Rather long tails. Multiple spurs on each legs, dark purple faces and a high degree of greenish luster on jet-black plumage	(Skinner and Hady 2018)
	Ameraucana	Black, Blue, Blue Wheaten, Brown Red, Buff, Silver, Wheaten, White	Cock: 6½ pounds Hen: 5½ pounds Cockerel: 5½ pounds Pullet: 4½ pounds	White	Blue/pastel	Dual purpose	Used mainly for eggs production	United States	Nice small pea comb and beards and muffs. Leg color is important	(Skinner and Hady 2018)

As a subdivision of breed, there is the variety: in this section there are different characteristics such as plumage color, comb type and the presence of beard or muffs. At the end there is the strains that are families or breeding populations sharing common traits. The strains are the results of one person’s or one organization’s breeding program (Skinner and Hady 2018). Not all the hundreds of chickens species are reported, informal or local breeds not officially recorded are excluded (Bett et al. 2014; Zindove et al. 2022).

This review describes the features of some particular chicken breeds. For each chicken breed is reported the use, as dual purpose or ornamental fowl. In Table 1 the average standard weight for each breed is reported, respectively for males and females. The first one is the Jersey Giant, the largest breed in the American class, with a weight of around 10–13 pounds, respectively for female and male. The smallest one is the Old English Game from England, with a weight of 4 pounds for female and 5 pounds for the male. Different types of chickens can also be identified on the basis of physical characteristics or temperament.

### Indonesian chicken species and description of Ayam Cemani chicken

Indonesia is one the most rich country as regard to the native chicken genetic resources. The growth of the breeding of native chickens in Indonesia is crucial. In fact, native chickens provide to more than 200 million Indonesian people requirement for animal proteins (Hidayat and Asmarasari 2015). In Indonesia there are at least 31 different types of native chicken breeds. In this section, the physical and the morphological characteristics of Indonesian indigenous chicken are described.

Ayam Cemani, also known as black chicken, is a very rare Indonesian local breed. As regard to the morphological characteristics, Ayam Cemani are completely black in color, with black plumage, comb and wattles, beak, eyes, skin and legs. Moreover, even the internal organs, bones and muscles are completely black (Łukasiewicz et al. 2015).

**Fig. 3:**
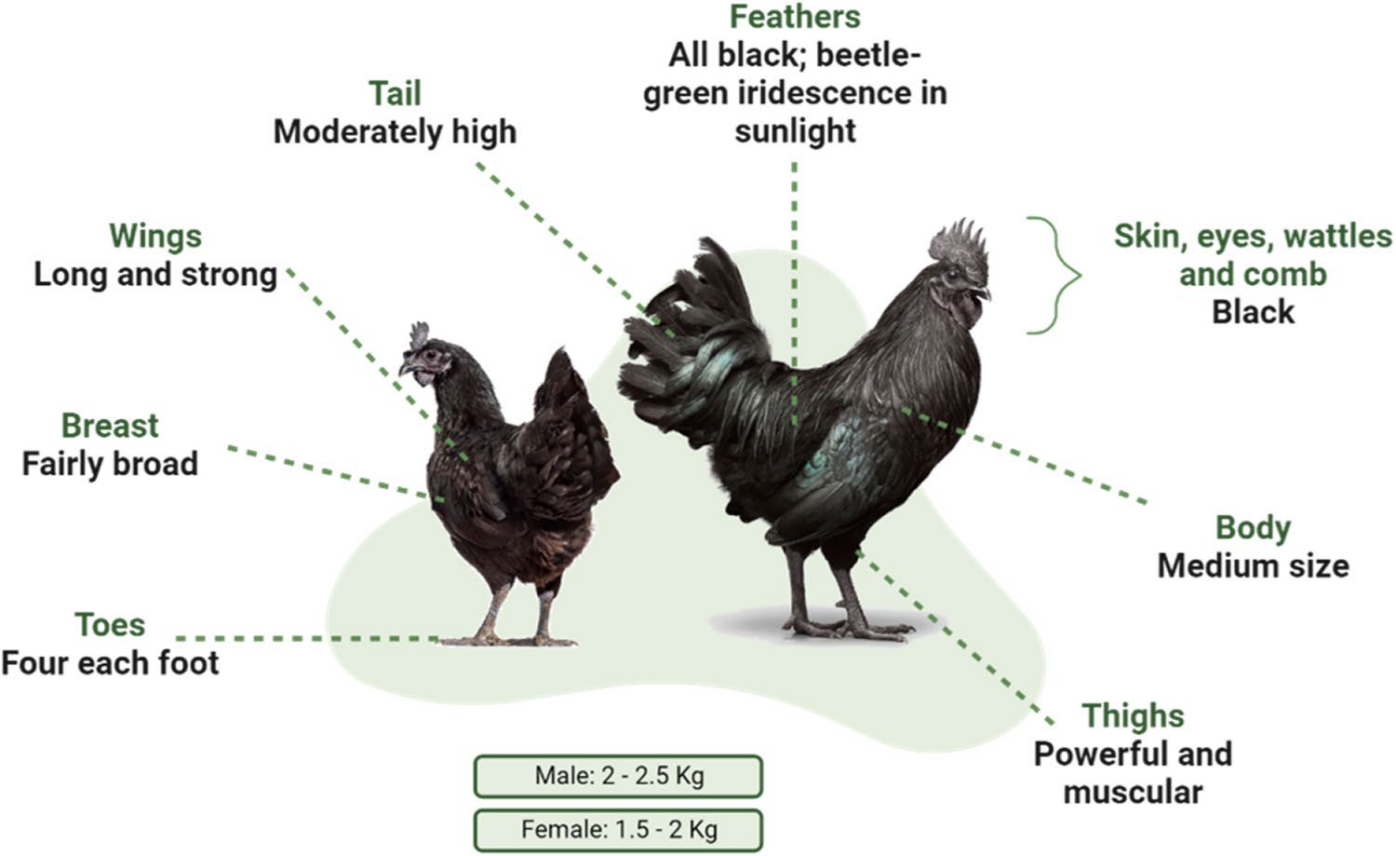
Physical and morphological features of Ayam Cemani breed

The hens lay eggs that are cream-colored and ranging in weight with an average of 45 g. Ayam Cemani hens are poor setters and seldom hatch their own brood (Fig. 3).

The average life expectancy of an Ayam Cemani chicken is up to 6 to 8 years. Despite this, lifespan can be prolonged if the right living conditions are applied. Ayam Cemani hens are in general calm and gentle, while the roosters are protective of their flocks. Typical behaviour is the fight for dominance of young roosters.

Ayam Cemani chicken has not an aggressive behaviour. In fact, it has also been reported that Ayam Cemani are quite sociable with not only other chicken breeds and other livestock but also with humans.

Sex determination of young birds is difficult and challenging before the development of the adult feathers.

Ayam Cemani are very hot tolerant chickens, as they are originated from a hot climate place such as Indonesia and for this, they perform better in hotter locations. Despite this, it is also possible to breed them in relatively cold temperatures, only if certain care is provided, such as a warm retreat. In particular, it is necessary to take care and protect Ayam Cemani comb during winters in order to prevent frostbite (Prakash et al. 2023).

Table 2 reports different types of indigenous chicken breed present in Indonesia. For each chicken breed is specified the distribution area and some of the most important phenotypical characteristics.

**Table 2:** Characteristics of Indonesian native chickens

Breed	Distribution area	Weight	Phenotype characteristics	Special characteristics	Use	References
Sentul	Ciamis West Java	Male: 1.5 – 3.5 kg Female: 0.8 – 2.2 kg	Main plumage colour is grey, which is its typical appearance. Skin color white to grey, single comb type	Higher growth rate and immunity to disease compared to other Indonesian species	Dual purpose	(Hidayat and Asmarasari 2015)
Pelung	Cianjur West Java	Male: 5 – 6 kg Female: 2.5 – 3.5 kg	The largest and the tallest body compared to other breeds. A grown male may stand up to 50 cm in tall	It is know for its long, melodious crow which make it one of the most expensive birds in Indonesia	Meat production and crowing contests	(Asmara et al. 2020, 2023)
Kampung	Found in most villages of Indonesian archipelago	Male: 2 – 2.5 kg Female: 1.5 – 2 kg	Both its physical characteristics and its coloring are highly variable. Three principal colour type: black-red variety, red type and naked-neck type	They are slow-growing breed that contributed to its low productivity	Dual purpose	(Hidayat and Asmarasari 2015)
Lamba	Southern Garut West Java	Male: - Female: -	It has a slightly larger body size than Kampung chicken, with long neck, single comb and longer crow compared to Kampung chicken	-	Dual purpose	(Hidayat and Asmarasari 2015)
Wareng	Indramayu West Java	Male: 2 – 2.5 kg Female: 1.5 – 2 kg	This small size native chicken	It is very alert and difficult to catch; it has better egg productivity than another Kampung chicken	Dual purpose	(Hidayat and Asmarasari 2015)
Banten	Banten	Both sex between 400 g – 1.1 kg	It has firm and compact posture, with short, small pea comb. It has also a strong neck structure while its plumage is very similar to that of Kampung chicken	Most suitable for small backyards	Eggs production	(Hidayat and Asmarasari 2015)
Ciparage	Karawang West Java	Male: 2 -3 kg Female: 1 – 2 kg	The male has a tall and solid body posture	It is now practically extinct	Dual purpose	(Saili et al. 2022)
Siem	Found around Bogor and Jakarta areas	Male: - Female: -	It has a shiny blue black plumage, it’s body size is slightly bigger than Kampung	It is well known to have an excellent mothering behavior	Meat production	(Hidayat and Asmarasari 2015)
Rintit/Walik	The Rintit can be found everywhere in Indonesia although in very small numbers	Male: - Female: -	This breed has a very distinctive appearance because it is plumage is frizzled. Very limited data are available on morphological features and current utilization	-	-	(Ulfah et al. 2016)
Nagrak	Nagrak Sukabumi West Java	Male: Female:	It has a much better growth rate than the Kampung and similar to Pelung. Nagrak chicken is raised to be sold as meat type bird	It is a cross breed of male Pelung and female Kampung which has 87.5% Pelung’s blood and 12.5% Kampung’s blood		(Hidayat and Asmarasari 2015)
White Kedu	Kedu Temanggung Central Java	Male: 2.2 – 3.6 kg Female: 1.2 – 2.7 kg	It has white plumage with big single comb on male’s head	The number of White Kedu is very small and it is impossible to gather a rather big number of White Kedu, within short time due to its rarity	Ritual birds	(Hidayat and Asmarasari 2015)
Black Kedu	Kedu Temanggung Central Java	Male: 2 – 3.5 kg Female: 1.8 – 2.7 kg	It is plumage is almost thoroughly black with big single comb	The hen lays more eggs than Kampung chicken hens	Ritual birds in Indonesia; Used as medicine in China	(Hidayat and Asmarasari 2015)
Sedayu	Sedayu Magelang Central Java	Male: - Female: -	It has a better size and body weight than Kampung chicken	It is also a good native layer, it is kept as egg producer at first year but after around 2 years production period will be sold as a good meat type native breed	Dual purpose	(Hidayat and Asmarasari 2015)
Cemani	Temanggung Central Java	Male: Female:	It Is Kampung or Kedu chicken which has a thoroughly black color of plumage, comb, wattles, tongue, skin, meat, leg scales and toes	Rare breed; The hens are poor setters and rarely hatch their own brood		(Hidayat and Asmarasari 2015)
Nusa Penida	Bali, Nusa Penida Island	Male: - Female: -	It has a small body size and very alert. The male has thick neck plumage, wide wings and considerably long tail feather, while the female has a nice crest on top of the head	-	-	(Hidayat and Asmarasari 2015)
Olagan	Bali	Male: 1.2 – 1.5 kg Female: 1.1 – 1.2 kg	It has no feather on the neck while its body form tend to looked wider than the Kampung. The plumage is very similar to that of Kampung chicken	They are also known as ornamental chicken because of their voice	Meat production	(Rofii et al. 2018)
Sumatera	Central region of Sumatera	Male: 2 – 2.5 kg Female: 1.8 – 2 kg	The male has a firm, compact and artistic body appearance with a long beautifully curved tail feather. Hens are poor layers	Strong flight ability	Ornamental fowl	(Hidayat and Asmarasari 2015)
Merawang or Merawas	Bangka Island South Sumatera	Male: - Female: -	The body plumage is not different from that of Kampung chicken	The male posture is big and firm with small short red pea comb	Dual purpose	(Hidayat and Asmarasari 2015)
Melayu	North Sumatera	Male: about 5 kg Female: about 4,1 kg	The body plumage is not different from that of Kampung chicken	One of the most ancient breed	Ornamental or showing fowls	(Hidayat and Asmarasari 2015)
Tolaki	South Sulawesi	Male: - Female: -	It has an upright body posture small head, long neck and back, supporter by a pair of long but strong legs. Its body plumage color is not much different from the Kampung	Very alert breed	Meat producers	(Hidayat and Asmarasari 2015)
Nunukan	Nunukan and Tarakan Island East Kalimantan	Male: 1,2 – 2,6 kg Female: -	The male has a rather tall and big posture while it’s female almost is similar size with the Kampung. Nunukan has a more uniform plumage color if compared with other breeds of native chicken. Male nunukan has an extremely short tail feather which is the typical characteristic of Nunukan breed	-	Dual purpose	(Hidayat and Asmarasari 2015)
Maleo	Central Sulawesi and Maluku Island	Male: - Female: -	The cock is tall and slender, with a big beak, blue faced and black crest on its head make it looked very attractive. Its plumage basically black and shinning reddish brown breast feather while it’s tail feather is white	-	-	(Hidayat and Asmarasari 2015)
Ayunai	Merauke Papua	Male: - Female: -	It has no feather on the neck and crop while its wattles are red and big. Its body appearance tend to be round	-	Meat producers	(Hidayat and Asmarasari 2015)
Jepun	-	Male: - Female: -	The size of this breed is smaller than Kampung chicken, the cock has a red single comb while the plumage color is about the same as that of Kampung chicken. One typical characteristic is that it has a very soft fluffy feather structure, looks like that the feathers are not grown well	-	-	(Hidayat and Asmarasari 2015)
Bangkok	All area	Male: - Female: -	It has a tall, wide and firm body with wide and strong wings, short red pea comb. The most common plumage color is black with some red combination on the neck, back, breast and wings for the adult male	Actually most of “Bangkok” chicken kept by the farmers is crossbred of pure male Bangkok with Kampung chicken	Dual purpose	(Hidayat and Asmarasari 2015)
Tukung	West Kalimantan	Male: - Female: -	Male and female Tukung has no tail feather at all. The body size usually smaller than Kampung chicken	It is possibly one of the rarest type of native chicken found in Indonesia	Ornamental fowls	(Hidayat and Asmarasari 2015)
Bekisar	Kangean Island, Madura, East Java	Male: - Female: -	It has a very attractive shining body plumage. Bekisar is a crossbred of male Green Jungle Fowl with domestic fowl. The plumage colour is very dependent on the on the parent’s plumage colour	The most highly appreciated native chicken in Indonesia. Its crow is very specific therefore it is known as singing bird. Very hard to find, most hens are sterile	Spiritual or symbolic mascot on outrigger canoes; crowing contests	(Hidayat and Asmarasari 2015)
Burgu	South Sumatera	Male: - Female: -	Brugo is very similar to dwarf chicken yet it has a slightly bigger body size	It is a crossbred of male Red Jungle Fowl with female Kampung chicken	Dual purpose	(Hidayat and Asmarasari 2015)
Kasintu	Its scientific name is *Gallus-gallus bankiva* lives mostly in northern part of Java, South Sumatera, Bali and South Sulawesi	Male: - Female: -	The main plumage color of the cock are black, ornamented with red color on its head, neck, back and waist. The hen usually has reddish brown plumage color with some blackish stripes	-	Dual purpose	(Hidayat and Asmarasari 2015)
Canghegar/Cukir/Alas	These wild birds live in southern part of Java, Madura and also in some other Indonesian Island	Male: Female:	It has much smaller body size than Kampung chicken. The body plumage of the cock basically black combined with shiny green scaly looked plumage. It has a big round rainbow colored single come with relatively long red wattles. The hen’s plumage color is pale brown with some small dark spots	These are the native names for Green Jungle Fowl in Sundanese, Madura and Javanese Languages		(Hidayat and Asmarasari 2015)
Balenggek	Solok West Sumatera	Male: 1.1 – 1.7 kg Female: -	It has three types i.e.,Gadang (big type), Batu (small type) and Ratiah (medium type). Their body plumage mostly combination of red, black and white	The hens crow like roosters	Dual purpose; Crowing contests	(Hidayat and Asmarasari 2015)

In comparison with other chicken species, both Cemani and Black Kedu and Balinese (Olagan) are characterized to be all black and all with black beaks. Nevertheless, in Balinese (Olagan) hens, black is not the dominant colour. Furthermore, the Ayam Cemani tongue is black, while that of the other breeds like Black Kedu, White Kedu and Balinese (Olagan) is grey. The eyes and combs of Ayam Cemani chicken are black. Instead, Black and White Kedu have red combs and wide black eyes, circled in orange. Again, the predominant color of the skin and feathers of Ayam Cemani is black, while Black Kedu, White Kedu and Balinese (Olagan) have pale and white skin. Moreover, black and white Kedu also have white neck feathers. On the other hand, Balinese (Olagan) is characterized by the absence of neck feathers. Ayam Cemani hens have the same shank color as Black Kedu hens, but Cemani hens have black coloured soles of feet, where as a Black Kedu hen have yellowish soles of feet. Instead, White Kedu and Balinese hens have yellowish legs and soles of feet. Thus, by comparing the characters of Ayam Cemani, Black Kedu, White Kedu and Balinese chicken breeds, from a phenotypic point of view, it can be stated that the Ayam Cemani showed the same quantitative phenotypical characteristics as black and white Kedu, but quite different from those of Balinese (Olagan) one. Moreover, Balinese (Olagan) has a taller and longer body, as compared to other mentioned breeds. In regard to the qualitative phenotypical characteristics of Ayam Cemani, it can be assumed that Ayam Cemani chicken differ from those of other breeds in body color, as their bodies are completely black (Rofii et al. 2018).

Compares to Silkie chickens, while both breeds showed an over accumulation of melanin, the overall phenotype of Ayam Cemani and other black chicken breeds, are completely different from the Silkie one. Indeed, the Ayam Cemani has black plumage and non-fluffy feathering. Instead, Silkie chickens has fluffy feathering. In addition to this, also the comb shape in Ayam Cemani males are completely divergent from that in Silkie males, which has rose combs Dharmayanthi et al 2017. Malaysia and Indonesia are two closely related countries. In Malaysia, native chickens are commonly known as Ayam Kampung (village chickens) and they are the outcome of a crossbreed between the Red Jungle Fowl and mixed exotic breeds imported by Europeans. Chicken breeds from Malaysia are enlisted in Table 3.

**Table 3:** Breed and characteristics of chicken breeds from Malaysia

Breed	Variety	Weight	Special characteristics	Main use in Malaysia	Other worldwide use
Ayam Kampung	Black-red, Red type, Naked-neck type	Male: 2 – 2.5 kg Female: 1.5 – 2 kg	Both physical characteristics and its colouring are highly variable Small, slow-growing breed; Eggs colour: white or light brown	Meat and eggs production	Pet Sacrificial offering in Indonesia
Maly Game		Male: about 5 kg Female: about 4.1 kg	Tallest breeds of chickens (over 90 cm in height). Five colour varieties: black, black-red, pile, spangled and white Eggs colour: brownish or golden	Meat production	Crossing to create new breed Showing
Serama	-	Both sex under 500 g	Upright posture, full breast, vertical tail feathers and vertical wings. Smallest breed of chicken in the world Eggs colour: tiny cream coloured	Meat and eggs production	Pet in United States

As regards a comparison of meat prices is concerned, in Indonesia, native chickens are more expensive than commercial hybrid chickens. This is due to the fact that local consumers prefer to pay more for Ayam Cemani meat, as it is of higher quality, tastier and lower in fat content. Moreover, also native chicken eggs are more expensive as compared to the commercial ones. This is because native eggs can be employed as part of the “Jamu”, the traditional herbal drink which is very popular in Indonesia. Ayam Cemani are also very expensive outside Indonesia, in the rest of the world. In fact, even in the United States, where Ayam Cemani are a still very rare breed, their cost is high. This is partly attributable to the fact that in US there is a ban on chicken imports from other parts of the world due to the Avian Flu Fever. Moreover, the black coloration gene is recessive and this leads to high incidence of color leakage. For this, perfectly black specimen of Ayam Cemani are hard to come by and consequently very costly (Sartika et al. 2011).

### Genotypical and phenotypical characterization of Ayam Cemani and main Indonesian native chickens

Till date, the use of Indonesian crowing chickens is increasing. For this, to assess their genetic structure is crucial to support the conservation of their genetic resources. Recently, some exotic breeds, characterized by a high performance in fighting ability, meat and eggs production or exhibition, have been imported from foreign countries. This could have an impact on the genetic diversity of native Indonesian crowing chickens. The genetic background of native crowing chickens is still unknown, it is important to notice their genetic origins in relation to foreigner chicken breeds (Asmara et al. 2023).

There are currently no studies available specifically discussing the genotype characteristics of Ayam Cemani or other Indonesian native chickens (Nganvongpanit et al. 2020).

Generally, phenotypical characteristics are the set of all those aspects, physical characteristics and properties that can be measured quantitatively and qualitatively. In order to apply the appropriate genetic program, it is crucial to identify the phenotypical characteristics of native chicken breeds. It is known that the phenotypical characteristics of chickens are determined by both genes and environmental factors (Rofii et al. 2018).

Native Indonesia chickens have particular phenotypical characteristics which are required to ensure the genetic diversity of the local chicken breeds and for the future development of a germplasm.

No differences in body height, body length and the length of tarsometatarsus were seen between Ayam Cemani, the Black Kedu and the White Kedu. Instead, a significant difference was observed between Ayam Cemani and Balinese (Olagan) breed (Rofii et al. 2018).

As regarding the origin of these native breeds, it has been reported that Ayam Cemani, together with the Black Kedu, the White Kedu and the Kampung chickens are all derived from a common ancestor, called the Red Jungle Fowl (*Gallus gallus*). It has also been reported that the genetic distance between these different chickens breeds can have an effect on phenotypical features such as the body length and size. For instance, Kedu chickens possess a higher genetic diversity and they are genetically distant from the other chicken types. It is known that the loss of genetic variability is determined by keeping the individuals in the same environment. This leads to the fact that there are no variation in characteristics like features of the beak, wing, femur and also the length of tibiotarsus. As the differences, even the variation of height, body length and tarsometatarsus length, observed in Ayam Cemani, Black Kedu, White Kedu and Balinese (Olagan), are also dictated by environmental factors. Moreover, it has also been observed that weather and environmental temperature such as 35 °C (noon) and 15 °C (night) could be the reason for the contraction of the Snot (Coryza) in some Ayam Cemani chickens. Chickens with this disease feed less, resulting in a negative effect on their growth (Ulfah et al. 2016).

### Ayam Cemani chicken and the mystery behind their black color

The Ayam Cemani is famous worldwide for its prominent features: the enterily black body. This condition, also known as dermal hyperpigmentation or Fibromelanosis, leads to the dark colour of the feathers, beak and also internal organs. In fact, most of their internal body parts, including also their blood, show dark black pigment deposition. More specifically, Fibromelanosis is a condition characterized by an intense pigmentation of the dermal layer of the skin, across the whole body, which leads to a dark blue appearance when viewed through the clear epidermis. It was given the name of Fibromelanosis to delineate the association between the dark pigmentation with the internal connective tissue. This can be easily seen in the trachea, pericardium, sheaths of muscles and nerves, gonads, blood vessels, mesenteries of the gut and bone periosteum (Dorshorst et al. 2011).

Usually, in chickens, only melanocytes are able to release pigmentation. Instead, in Ayam Cemani chicken, in which Fibromelanosis is reported, all kind of cells over melanocytes are able to release pigment and there is an accumulation of melanin in the internal organs and connective tissue.

The phenotype Fibromelanosis (*Fm*) can be also observed in other domesticated chicken breeds than Ayam Cemani, such as Kadaknath in India, Black H’Mong in Vietnam, Silkie from China, Argentinean Tuzo type in Argentina and Svarthona in Sweden. Among these species, Ayam Cemani breed exhibits Fibromelanosis condition similar to Chinese Silkie chicken. This is due to the presence of the *endothelin 3* gene (*EDN3*) which was in duplicate on chromosome 20. In a study conducted by Dharmayanthi et al. (2017), they showed that Fibromelanosis (*Fm*) in Indonesian Ayam Cemani and Chinese Silkie is the result of the same genetic change, involving the *EDN3* duplication on chromosome 20. It is now known that this genomic single origin change arises spontaneously in the ancestral populations of Red Jungle Fowl in Asia. It is also known that probably this happened well before the domestication of chickens. Then, a strong artificial selection to tick the *Fm* phenotype is clear in the genetic variability near the target site of duplicated *EDN3*. Nevertheless, there is a sensitive variability in the genetic pattern of Ayam Cemani and Silkie Chinese breeds, probably due to the domestication process Dharmayanthi et al 2017. In Fibromelanosis (*Fm*) phenotype, mutation occurred in the *Fm* gene leads to the accumulation of melanin pigment not only in the skin but also in other organs (e.g. blood vessels, muscles, tracheas and gonads). This leads to suggest that the *Fm* traits could not be enterily fixed in the population. Recently of concern is the fact that, due to the no fixation of *Fm* traits, changes are occurring in Ayam Cemani population on the most distinguish feature.

In order to identify the causes of the black skin color molecular studies have been conducted in domestic chickens. More recently, through the use of advanced genetic methodologies, it has been possible to identify a genomic region in chromosome 20 including *EDN3* and *BMP7*, which are associated with the characteristic hyperpigmentation in chickens combs (Dong et al. 2019). In their study, Dong et al. (2019) highlighted, via PCR and PCR–RFLP, the different *Fm* genotype presents currently in the Ayam Cemani chicken population. In fact, the majority of Ayam Cemani with deep black comb phenotype showed the *Fm*/*Fm* homozygote genotype. On the other hand, some Ayam Cemani individuals with reddish black combs, exhibited an heterozygous genotype. They also wanted to elucidate why some individuals possessed a brighter skin color and more reddish black combs. They also found that some Ayam Cemani with reddish black combs were *Fm*/*Fm* homozygous (Dong et al. 2019). Nowadays, Ayam Cemani is bred by or in small farms or by individual breeders, principally in the Kedu village (Central Java). In some cases, some breeders mate Ayam Cemani breed with other breeds or other Kedu chickens. For this reason, some Ayam Cemani individuals have an incomplete *Fm* traits.

### The first eggs laid by the of Ayam Cemani chickens (known as Tembean) and their significance in the Indonesian culture

The first egg laid by each Ayam Cemani chicken is named “Tambean”. These eggs are quite rare and they have a great importance in the Indonesian culture. In fact, there is a mystical belief that their consumption can help those who eat it to conceive a child. In general, despite Ayam Cemani eggs are very valuable and high in cost, breeders give away the Tambean egg for free either to couples in need or to healers. The reason is that in Indonesia much importance is given to the offspring. The healers usually roast the Tambean egg until it becomes a fine powder. Then, they mixed it with honey and serve to drink in one shot for both the wife and the husband. It is important to consume it immediately after its preparation.

## Farming Ayam Cemani breed

Ayam Cemani raised in Indonesia, their country of origin, are used for eggs and meat production in order to reach the domestic market needs. Instead, specifically in Java island, Ayam Cemani are used in ritual ceremonies and in folk medicine for the treatment of cardiovascular and respiratory diseases. Ayam Cemani are considered as gamecocks for game fighting in Bali, where this is considered as an age-long cultural tradition and an ancient religious ritual rather than a sport or an entertainment. On the other hand, in Europe, Ayam Cemani are raised as ornamental chicken breed (Łukasiewicz et al. 2015).

The largest collection of Ayam Cemani all around the world is kept by the Congolese-Belgian philanthropist Jean Kiala-Inkisi in Africa. Ayam Cemani chickens are part of a breeding program carried out by the African Ornamental Breeders Association (AOBA) in Kenya and in the Democratic Republic of the Congo. In particular, Ayam Cemani chickens belonging to the collection of Kiala-Inkisi are divided into three strains: the first one, the European strains (mainly Belgium/Netherlands bloodlines). The second one, the Javanese fighting line, in which all roosters are kept separated in a leg-hitches or sometimes they are kept in a ratio of 1:10 rooster: hens in separated holding pens. The third one, which is the Indonesian/Javanese regular farm type. They are kept in free range with a ratio of 10:100 rooster: hens. Moreover, they sleep in fig trees. At the moment the Ayam Cemani present on the collection are 500 roosters and over 1000 hens. Ayam Cemani chickens are fed with a mix of edible insects, together with rice, broken maize or sorghum. The price at which they are sold varies from 2500 euro/rooster (Asmara et al. 2023).

At the moment, in Indonesia, the number of household of native chickens farmer is equal to 20.851.901. It can be assumed that the main centre of native chickens farmer household is in the Java Island as shown on Table 4. (Hidayat and Asmarasari 2015).

**Table 4:** Percentage of Ayam Cemani Chicken farm divided by province in Indonesia

% of Native chicken farmer household	Province	Reference
21.75	East Java	(Hidayat and Asmarasari 2015)
20.84	Central Java	(Hidayat and Asmarasari 2015)
15.02	West Java	(Hidayat and Asmarasari 2015)
4.87	Lampung	(Hidayat and Asmarasari 2015)
4.39	Southern Sulawesi	(Hidayat and Asmarasari 2015)
4.18	North Sumatera	(Hidayat and Asmarasari 2015)
3.81	Banten	(Hidayat and Asmarasari 2015)
2.76	Southern Sumatera	(Hidayat and Asmarasari 2015)
2.64	East Nusa Tenggara	(Hidayat and Asmarasari 2015)
2.23	Yogyakarta	(Hidayat and Asmarasari 2015)
2.15	West Nusa Tenggara	
1.88	West Kalimantan	(Hidayat and Asmarasari 2015)
1.87	Bali	(Hidayat and Asmarasari 2015)
1.60	West Sumatera	(Hidayat and Asmarasari 2015)
1.58	Riau	(Hidayat and Asmarasari 2015)
1.15	Southern Kalimantan	(Hidayat and Asmarasari 2015)
1.03	Jambi	(Hidayat and Asmarasari 2015)
0.83	Southest Sulawesi	(Hidayat and Asmarasari 2015)
0.74	Central Sulawesi	(Hidayat and Asmarasari 2015)
0.69	Bengkulu	(Hidayat and Asmarasari 2015)
0.68	Central Kalimantan	(Hidayat and Asmarasari 2015)
0.61	Papua	(Hidayat and Asmarasari 2015)
0.57	East Kalimantan	(Hidayat and Asmarasari 2015)
0.44	North Sulawesi	(Hidayat and Asmarasari 2015)
0.40	Jakarta	(Hidayat and Asmarasari 2015)
0.38	Bangka Belitung	(Hidayat and Asmarasari 2015)
0.36	Gorontalo	(Hidayat and Asmarasari 2015)
0.27	Maluku	(Hidayat and Asmarasari 2015)
0,14	North Maluku	(Hidayat and Asmarasari 2015)

**Fig. 4:**
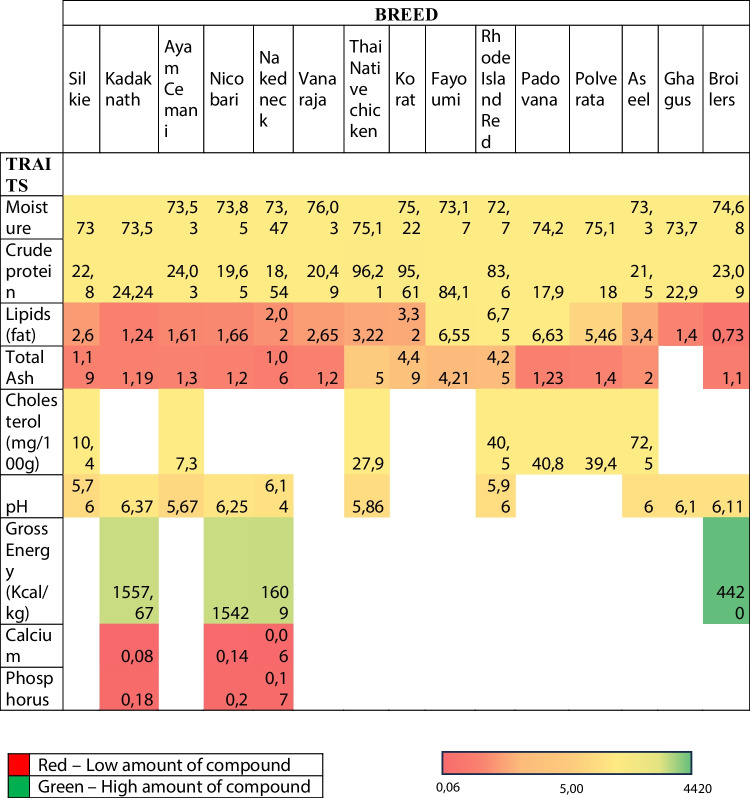
The heat map shows a comparison of the nutrient meat composition (expressed in %) between different poultry breeds, including the Ayam Cemani. The colour scale shows the trend in nutrient composition in the various species: red corresponds to the low amount of nutrient and green to the highest amount of nutrients

Native chickens have always been considered as an essential part of traditional rural living. Generally, in a farm household around 10–20 birds are kept. In Indonesia, more than 70% of the national consumption of meat is derived from poultry. Moreover, indigenous chickens, as Ayam Cemani, which are usually raised in rural area, have several potentials in order to support the national meat consumption. In fact, they are characterized by having a superior taste and a higher quality of the meat. In addition to this, the price of meat of Ayam Cemani is considered to be stable and even going up. This makes the breeding of Ayam Cemani by farmers in rural areas advantageous in order to increase their income (Wantasen et al. 2014). Ayam Cemani chickens are in general employed in folk medicine for their medicinal properties, mainly caused by Fibromelanosis. In a study conducted by Kostaman et al. (2021), they reported the same amount of saturated fatty acid (SFA), monounsaturated fatty acid (MUFA) and poly-unsaturated fatty acid (PUFA) profiles in egg yolks and albumen from Ayam Cemani and White Leghorn chickens. It is also known that the cholesterol level present in an egg is about 218.12 mg/100 mg. For these reasons, Ayam Cemani meat and eggs are reported to be favourable to others chicken derivates by health-conscious people. Moreover, it has been reported by Sehrawat et al. (2022) that the amount of proteins found in Kadaknath chickens breast muscle was 25.25 ± 0.31 (g/100 g of tissue) and this value was slightly lower that the one founded in Ayam Cemani chickens. At the same time, the protein content of thigh muscle (19.98 ± 0.29) in Kadaknath chicken is slightly lower than that of Ayam Cemani (21.4 – 21.6%). On the other hand more older studies, carried out by Michalczuk et al. (2014) reported a fat content of 0.73 – 1.03% in Ayam Cemani chickens in the breast muscle, same value measured in Kadaknath, and a higher level of fat content (3.6 – 3.7%) in the leg muscles. The fact that the black meat of Ayam Cemani is characterized by a lower fat content, makes it suitable for the diet of people suffering from high blood pressure. In conclusion, taking together the reported results suggest that poultry derived products from black chickens as Ayam Cemani have a superior quality due to the lowest value of cholesterol and lowest lipid content comparing with other chickens breeds (Bhat et al. 2015; Haunshi et al. 2022; Suyatno et al. 2023) (Fig. 4).

**Fig. 5:**
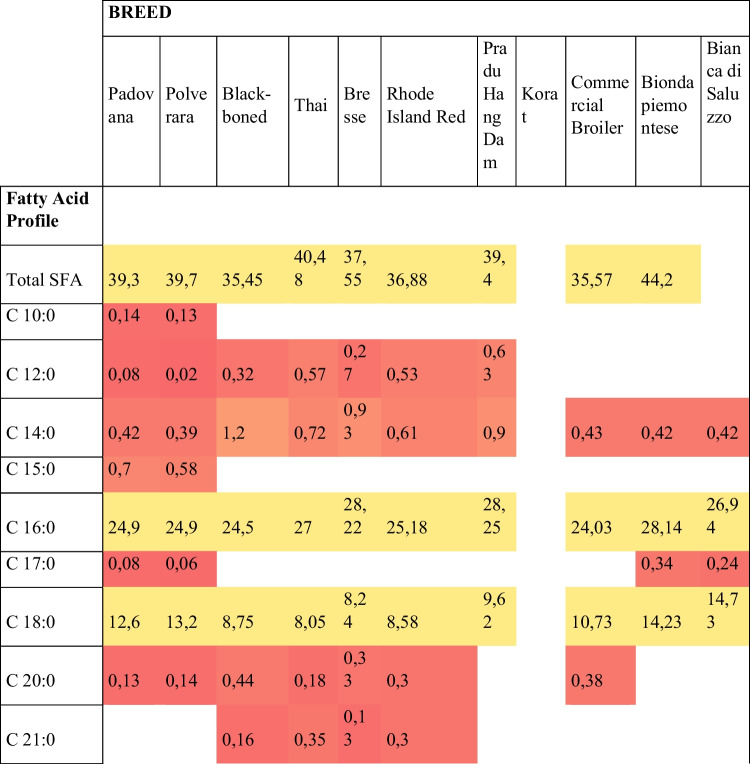

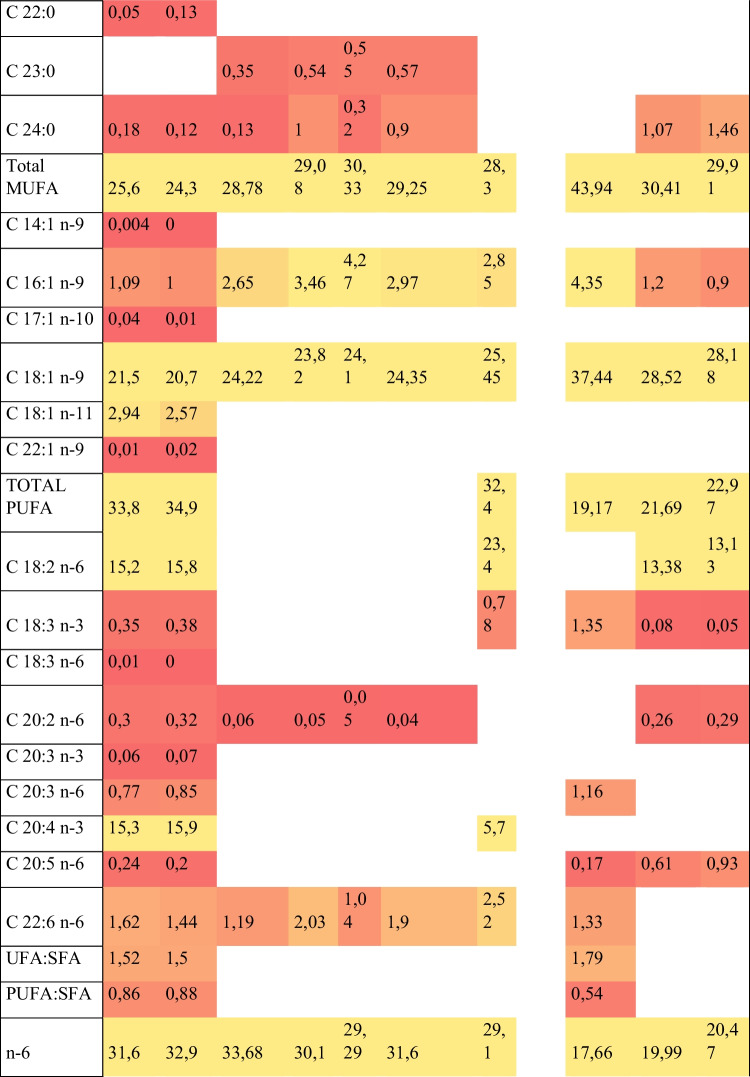

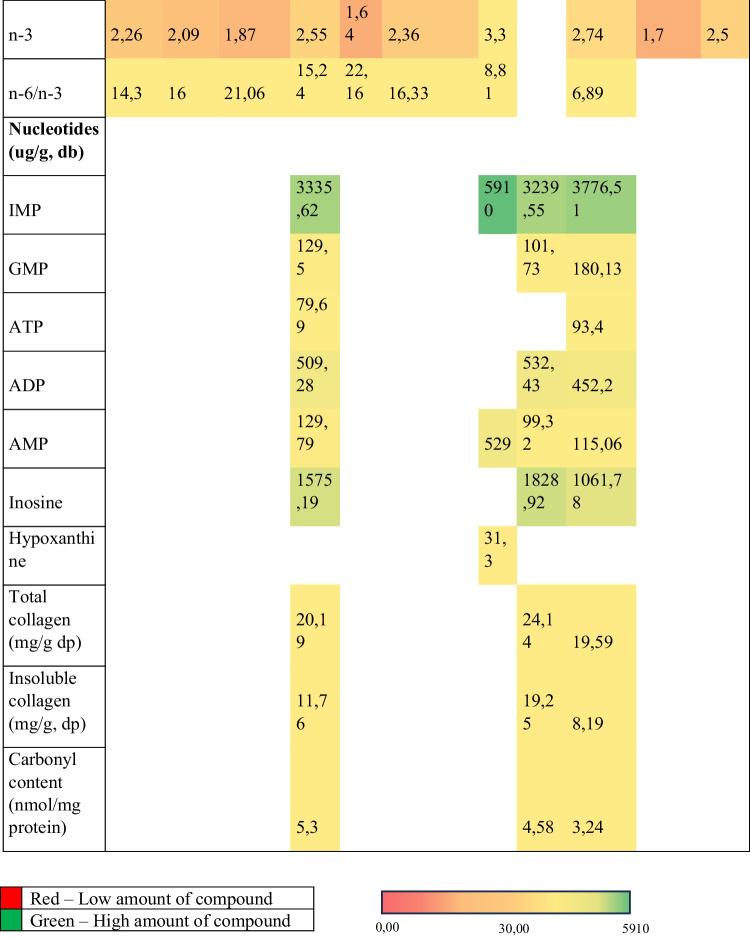
The heat map shows a comparison of fatty acid and nucleotide profile between different poultry breeds. The colour scale shows the trend in compounds composition in the various species: red corresponds to the low amount of compounds and green to the highest amount of compounds

In Fig. 5 is reported instead a comparison in fatty acid and nucleotide profile between different poultry species.

Instead, no differences in meat or eggs composition were founded in Ayam Cemani between females and males. As regard the fibre diameter in breast and leg muscles, it was highlighted these are smallest in Ayam Cemani chickens when they are compared to other chickens breeds. The values reported for fibre diameter in Ayam Cemani chickens were 45.06 μm for female and 47.08 μm. Also for leg muscles it has been observed the same trend, with value ranging in size for female Ayam Cemani around 36.30 μm and for male around 39.09 μm. A smaller diameter of muscle fibres is beneficial for meat juiciness. Instead, if there is the presence of a higher proportion of large-diameter muscle fibres, these leads to a significant increase in the cooking loss.

In Ayam Cemani was reported the lowest cooking loss of the meat during cooking process both in breast meat and in leg muscles. Furthermore, the muscles of Ayam Cemani chickens were found to be high in water-biding capacity compared with the other chickens breeds. On this, genotype as a significant effect, determined as forced drip loss, which was lower in Ayam Cemani chickens (Prakash et al. 2023). As reported by Łukasiewicz et al. (2015), the water-holding capacity is a pivotal indicator of meat technological quality. In Indonesia, Ayam Cemani is now at risk of extinction due to the overconsumption of their meat and an inappropriate approach in genetic preservation and proliferation. With the aim to solve this problem some preservation and breeding program have been started from several different agencies (Prakash et al. 2023).

The Greenfire farms located in Florida (USA) is another famous breeding farm for Ayam Cemani chicken breeds all around the world. In this farm, they carried out a process of improving bloodlines, creating genetic diversity and ensuring that this particular breed can potentially survive in American states. They carefully evaluate and select the Ayam Cemani offspring for their inclusion in breeding programs. This process consists in a series of expensive and time-consuming steps which allows to establish and improve the breed. Ayam Cemani chicken breed is held in large and well-built pens or kept in free-range breeding system. The pens in which are holding Ayam Cemani chickens are large, airy enclosures with no solid walls or boxes. In this way, Ayam Cemani chickens are maintained healthy, hardy and acclimated to natural temperatures. Moreover, to be sure to reduce the potential risk of infectious disease, visitors are not allowed to enter in the farm. As regard to the feed, in the Greenfire Farm, they supplement their foraging diets with high-quality feeds. In addition, there is an ongoing process to enhance the creation of new techniques and the fabrication of custom equipment for the implementation of raising these kinds of chickens breed (Pitesky et al. 2019).

In United States, Ayam Cemani is deemed an ornamental chicken breed and they are still very rare. Indeed, the number of Ayam Cemani reported worldwide is supposed to be below 3500 heads. This makes Ayam Cemani one of the most rare chicken breeds present all over the world (Nguyen et al. 2023).

**Fig. 6:**
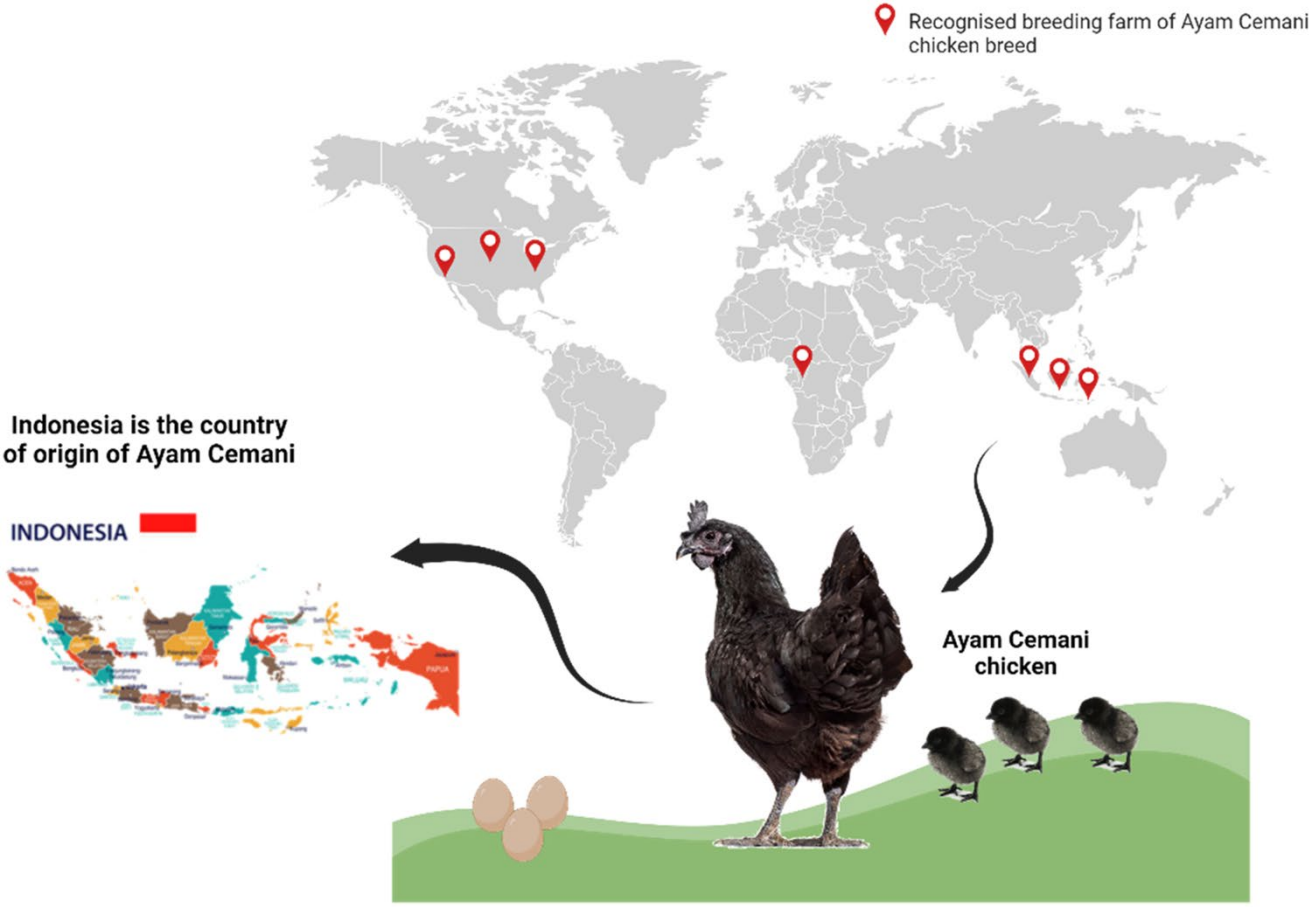
Figure depicts the recognised breeding farm for Ayam Cemani all over the world

This leads to an incrementation of the price and tends to make Ayam Cemani chickens quite expensive. For instance, Cackle Hatchery (Missouri, USA), an hatchery specialized in the commerce of rare chicken breeds, sells unsexed Ayam Cemani baby chicks at 49$ per chicks or Ayam Cemani hatching eggs for 14$ for egg. Moreover, in United States, Ayam Cemani is not considered an heritage breed and it is not recognized by the American Poultry Association. Also in Europe, Ayam Cemani chicken breed is considered as an ornamental chicken breed. Till date, the Ayam Cemani chicken breed is kept in the following European states: Netherlands, Belgium, Germany, Slovakia, Sweden and at least Italy and Czech Republic (Fig. 6) (Suyatno et al. 2023) (Fig. 6).

## Breeding systems for Ayam Cemani breed

In 1970s the majority (80%) of indigenous chickens were reared by traditional system and on each farm there were around 30 heads. Instead, starting from 1980s up to now, in Indonesia it is possible to find three different rearing system for native chicken: the traditional, the semi-intensive and the intensive one. It is also known that the transition between the traditional rearing system to the semi-intensive or the intensive one has made a positive effect on the local chicken production. It was demonstrated by Hidayat and Asmarasari (2015) that through the application of the intensive rearing system, there was an improvement in quality the native chickens. Table 5 provides the description of the different farming models, which are also explained in the detail below.

**Table 5:** Advantages and disadvantages using three farming models to breed Ayam Cemani Chicken

Type of farming model	Adventages	Disadventages	Reference
Traditional type	No cost for chickens’ maintenance, for eggs/meat production; Profit from selling is absolute	No suitable for increasing native chickens productivity	(Hidayat and Asmarasari 2015)
Semi intensive production system	Possibility to observe daily the growth of poultry; Possibility to achieve an higher production of meat and eggs. Reduction in the production cycle	Increase cost for poultry management and for the feed supplementation	(Hidayat and Asmarasari 2015)
Intensive production system	Maximum of productivity	Increasing costs for cages, feed, water, feed supplementation and medicines. Needing of expert farmers	(Hidayat and Asmarasari 2015)

### Traditional type

In the traditional type, native chickens are reared from first day until their death freely and without the intervention of farmers. In this case, feed is not provided, there are not cages, no application of health management or technology implementation. They sleep at night on tree around the farmer’s house. Usually the number of native chickens kept in this system is from 2 up to 20. There is no cost for the maintenance, or for the eggs or meat production by native chickens in the traditional rearing models. The profit income from the selling of chickens reared in this system is absolute for the farmer. This traditional model is the most common rearing system used in the countryside and this is due to the fact that most farmers have no capital or access to financial institutions to buy feed, supplements or medicine for their animals. On the other hand, due to the inability to control native chicken feed consumption, this breeding system is the least suitable for native chickens to increase their productivity (Hidayat and Asmarasari 2015).

### Semi-intensive production system

In the semi-intensive breeding system, Ayam Cemani chickens are housed in an open-fenced area, generally built in the backyard of the farmer’s house. Medical treatment are not routinary whereas feed and drink are provided regularly by the farmer. In the majority of cases no cages are provided to sleep at night and the native chickens sleep anywhere around on the farm. The average numbers of head of native chickens usually housed in this breeding model vary from 25 up to several hundred. They are generally breed not for commercial purposes. Moreover, generally no technologies are employed in this semi-intensive breeding system. Also according to Mengesha (2012) the semi-intensive one is a farming model in which native chickens are raised in small fence space with a feed supplement routine. In this system it is possible to observe daily growth of poultry. In addition, in the semi-intensive farming method is it possible to achieve an higher production of meat and eggs more efficiently compared to the traditional method and this leads to ensure animal protein and food availability for rural communities (Hidayat and Asmarasari 2015).

Wantasen et al. (2014), conducted a study in which they aimed to determine the income and the factors that have an impact on the semi-intensive breeding model for native chickens. It is thought that the semi-intensive model is a better system to breed native chickens and in particular Ayam Cemani, compared to the traditional one. It has been proposed a model for semi-intensive breeding model, called “Family Poultry based on semi-intensive chicken farming” in which was evaluated the food availability from animal protein with 10 hens and 1 cock per household. After the program first year, the amount of native chickens were increased up to 5.8 times and farmers family was able to benefit of 150 eggs and 24 native chicken meat. As compared to other near countries, such as Malaysia or Thailand, meat consumption in Indonesia is lower. Some studies have demonstrated that the semi-intensive breeding system is one of the most valuable method to increase not only the production but also the productivity and the income in small holder farming. In fact, in semi-intensive approach, the production cycle of Ayam Cemani chicken took about 59 days comparing to the 158 in the traditional one. This result indicates that the semi-intensive method applied in chicken farming could reduce production cycle and at the same time increase the egg production (Wantasen et al. 2014).

### Intensive production system/Ameliorate type (i.e.battery cage)

In the intensive breeding system the chicken population is divided in 3 groups based on their life period. In this model, native chickens are breed in cages and feed, water, feed supplements and medicines are regularly provided by the farmer. The production is generally done for a commercial purpose and it is completely business-oriented. Farmers are usually very experienced, with a wide network and pay attention to maximize the efficiency and productivity of their native chickens. The numbers of chickens kept in intensive model is from hundreds to thousands heads, according to financial sources (Hidayat and Asmarasari 2015). The cost for cages and equipment used in intensive farming model is about IDR 267,455 for year (Wantasen et al. 2014).

The Ayam Cemani itself is a breed characterized by a higher resistance to diseases, especially viral infections and to pathogens of intestinal tract. Moreover, further studies are needed to totally explain the potential of this Indonesian breed (Kostaman et al. 2021).

## Microbial contamination in chicken meat from Indonesian local markets

In general, chicken meat, due to its physical and chemical characteristics, is highly perishable. In fact, the great presence of nitrogenous compounds, lipids, carbohydrates and vitamins, and its water holding capacity leads to the creation of an environment suitable for the growth of microorganisms, which can alter meat quality. Several different pathogenic microorganisms have been identified in poultry meat, like as *Salmonella* spp., *Campylobacter* spp., *Staphylococcus aureus*, *Escherichia coli* and *Listeria* spp. Most of these bacteria species have a zoonotic significance and therefore they can have important repercussions on both Public Health and the economic sectors (Wardhana et al. 2021).

In a study carried out by Wardhana et al. (2021), it has been reported that there was microbial contamination in several chicken meat samples taken from local markets in Indonesia. The major contaminations found were caused by the presence of *S. aureus* (6.7%), *Salmonella* spp. (85%) and *E. coli* (90.03%). In addition, they showed that the prevalence of *S. aureus* (58.3%) detected in chicken meat from 10 Indonesian markets was higher comparing to that found in raw chicken meat sold in Ghana (9.2%). Twenty nine out of 60 chicken meat samples examined in the present study, resulted positive for *Salmonella* spp. strains (48.3%). This prevalence was higher compared to the one registered in other countries, such as Ghana and China (Wardhana et al. 2021). Also regarding the *E. coli* contamination, in this study it was reported a percentage of 96.7% of this bacterium in poultry meat from Indonesian local market. Together these results indicate that the chicken meat from local markets is unsafe for human consumption, based on critical control point limits. For this reason, could be desirable to implement the hazard analysis critical control points system and the food hygiene practice during food handling and processing (Wardhana et al. 2021).

## Feeding management of Ayam Cemani chickens

With the aim of achieving an optimum performance, in terms of expression of genetically improved breed, it is necessary to have an appropriate feeding management with particular attention to the nutrient requirements. In this way it is possible to enhance the biological and economic efficiency. In a study conducted by Applegate and Angel (2014), they reported that there is the will in the scientific community to start to study how to improve the nutrients commonly used for the compositional growth and also egg production (Hidayat et al. 2017). It is known that in hens the feed consumption is one of the parameters that influence the entire body metabolism and the ossification of the bones. In their work they highlighted that there were not differences in the average body weight of Ayam Cemani, Black Kedu, White Kedu and also Balinese (Olagan) chickens due to the fact that all breeds considered eaten the same kind of feed and were held under same environmental conditions. It is also well known that an increase in the proteins amount of the feed enhances the chicken’s growth. At least, even if all chicken breeds fed with the same diets demonstrated to have a similar and comparable body weight. Ayam Cemani resulted to be the more efficient in the utilization of the nutritive compounds present in the feed. As regarding the feeding for native chickens, raw materials such as rice bran, dried rice and corn are mainly obtained from Indonesian local market. As showed in the study, the feed consumption has the higher impact on the cost of production in the semi-intensive farming system. In fact, the average cost per feed for year is equal to IDR 9,175,564. This is due to the fact that farmer who gave 100% broiler feed to Ayam Cemani chicken 100% broiler feed on 0–30 days old chicks and decreased until 20% step by step on 61–75 days old whereas the feed made by rice bran and dried rice/corn is up to 40% (Rofii et al. 2018).

As regarding the feeding, like other animal species, poultry also need the right proportion of proteins, carbohydrates and fats, supplemented with minerals and vitamins. For instance, Ayam Cemani hens laying eggs need to be feed with food with 18.8% of protein. Moreover, the addition of grit to aid digestion and crushed oyster shell to reinforce the eggshell is desirable to a good health and egg production. Ayam Cemani diet depends strictly also on the breeder. For example, they add special supplement to improve flock’s intestinal health. Commonly additives protein such as dried Soldier Fly Larvae are administered to chickens (Saili et al. 2022). In Indonesia, Ayam Cemani are fed twice a day adding papaya and taro to increase their appetite and boost the immunity (Saili et al. 2022).

In a study published by Prakash et al. (2023) it was evaluated the feed conversion ratio between Ayam Cemani, Silkie and Kadaknath chickens. The feed efficiency derived from the union of the efficacy of metabolism together with the capacity to adapt in the local environment. All these breeds taken in consideration for the study results to possess a low feed efficiency if it is compared with commercial broilers and layers.

The results of the comparative study showed that the feed conversion ratio was higher in Ayam Cemani and Silkie chickens then the Kadaknath. Other study reported that the feed conversion ratio of Ayam Cemani was equal to 4.1, in a rearing period of 18 weeks (Prakash et al. 2023).

## Economic value of Indonesian native chickens

Asia Pacific’s position in poultry production is higher than the other countries and it has reached 27.37% in 2018. While Indonesia is in the 10th place of meat production in poultry in the world (Labour and Organization 2014). In addition, except for meat and egg production, some chicken species are also related with religious ceremonies, such as Cemani and White Kedu Chicken (Hidayat and Asmarasari 2015). Cemani Chicken is commonly used for traditional ceremonies, such as clean the village, “Sekatenan”, and “Grebeg Maulud” (Putri Syifa Camilla et al. 2023). In those sacred events Cemani Chicken will be an offering to the ancestors. Cemani Chicken is believed to have magical powers to repel evil deeds committed by spirits. Not only in traditional ceremonies, in some construction process and also business, such as the groundbreaking ceremony for building bridge or the opening ceremony for a new office, Cemani Chicken is always present, as the goal is to make the construction and business process run smoothly and safely (Putri Syifa Camilla et al. 2023). Table 6 shows which chickens are generally consumed by household consumers in Indonesia. There are at least five chicken types intended for restaurants, namely broilers chicken, Native Chicken (Ayam Kampung), Pelung Chicken, Sentul Chicken, and Cemani Chicken. Broiler chickens are not originally from Indonesia, but they are the most consumed chicken meat in this country. People in Indonesia are very familiar with broiler chicken meat since it is an affordable and tender meat (Hidayat and Asmarasari 2015). Apart from being raised for meat, broiler chickens are sometimes also raised for the eggs. The price of broiler chicken’s egg is also cheaper than the other chicken species’ eggs. (Marlina et al. 2015; Nuswantoro 2020). The comparison between broiler chicken meat and eggs and the other chicken meat species around the world shown on Table 6. Native chickens (Ayam Kampung) have a long development story in Indonesia. Initially, it was the chicken living in the forests, then later it was domesticated and developed by the communities in rural areas (Achmad 2010). Free-range chickens are native chickens adapted to Indonesia’s tropical environment. Rural society maintains it as a source of family food for eggs and meat (Kestaria et al. 2016). The chickens experienced natural selection and spread or migrated with humans and were then cultivated for generations until now (Hadi et al. 2021) The term native chicken was originally the opposite of the term broiler chicken, and this designation refers to chickens found roaming freely around housing area (Munir et al. 2016). However, since a program for developing, refining and breeding several superior local chickens has been carried out, several superior native chicken breeds have now become known and widely used as meat sources. To differentiate between them, there are now known terms free-range chickens (non-breed chickens) for native chickens that have been selected and maintained by improving cultivation techniques (not just showing off and left to find their own food) (Munir et al. 2016). Village chicken farmers have a role which is quite large in supporting the economy of rural communities because has high adaptability to the environment and its maintenance is relatively easier (Suprayogi et al. 2018).

**Table 6:** Distribution and uses of chicken breeds in Indonesia

No	Chicken breed	Origin	Aims of distribution (household/trading/restaurant)	Aims of utilisation (breed/meat/egg)	Trends (Decrease/Increase)	Reference
1	Broiler chicken	-	Household and restaurant	Meat and eggs	Increased	(Hidayat and Asmarasari 2015)
2	Native chicken (Ayam Kampung)	Java	Household and restaurant	Meat and eggs	Increased	(Hidayat and Asmarasari 2015)
3	Pelung Chicken	Cianjur	Trading	Breed	-	(Asmara et al. 2023)
4	Sentul Chicken	Ciamis	Household	Breed, meat, and eggs	-	(Hidayat and Asmarasari 2015)
5	Cemani Chicken	Temanggung	Trading and household	Breed, meat and eggs	Increased	(Hidayat and Asmarasari 2015)

There are many free-range chickens, also known as “free-range chickens uses and benefits to support human life”, among others, and their maintenance is very easy because they are resistant to environmental conditions, poor management, do not require a large area of land, can be done on surrounding land house. The selling price is stable and relatively higher compared to chicken other broilers and they are not easily stressed by rough treatment. Moreover, their body resistance is stronger compared to other broiler chickens (Hadi et al. 2021). Apart from these advantages, free-range chickens also have them several weaknesses, including the difficulty of obtaining good seeds and lower egg production compared to layer chickens and their growth is relatively slowly. For this the maintenance time is longer, especially this situation caused by low genetic potential. Those factors make the price of Ayam Kampung meat and eggs higher as compared with broiler chicken (Hadi et al. 2021).

Pelung Chickens are local Indonesian chickens that are raised for their unique voice and usually used for trading and crowing contest (Partasasmita et al. 2017). In general, Pelung Chicken has long and rhythmic vocalization that also stands apart from other local chickens due to its unique body type. Adult male Pelung Chickens can weigh up to 5,400 g, but adult females can only weigh 4,500 g. A total 39–68 eggs can be produced annually by the chickens (Asmara et al. 2020). Pelung's plumage lacks any distinguishing color or pattern. Nonetheless, male chickens typically have red, black, and green plumage, whilst females typically have black plumage (Partasasmita et al. 2017). According to a study by Asmara et al. (2019a), the two main colors of the plumage on male Pelung Chickens were red and black. The majority of female Pelung Chickens have plumage that is either black or yellow–brown (Asmara et al. 2019b).

According to ministry of agriculture officially (2011), Pelung Chickens are considered as an indigenous chicken breed in Indonesia that needs conservation because of its rarity. Pelung Chickens are threatened because their population are reduced. Consuming Pelung Chickens are also not common because this chicken raised for their crow (Asmara et al. 2020).

Sentul Chickens have grey with a little red-golden feathers color (Mugiyono et al. 2015). Sentul Chickens have a good potential to be meat and egg producers due to their good performance in terms of productivity (Depison et al. 2020). According to Mugiyono et al. (2015), Sentul Chickens tend to be superior to other local chicken in a number of ways, including comparatively quick growth and greater egg output than native chicken (Ayam Kampung). The presence of these advantages, make Sentul Chickens as an alternative industrial commodity. However, the information about Sentul Chickens in Indonesia recently is not widely known and generally only used in some specific areas such as its origin place, Sentul city (Hidayat and Asmarasari 2015).

In general, Ayam Cemani chickens are not only intended to be kept as ornamental chickens but also reared to produce eggs which will later be hatched. Cemani Chickens have all black body features, start from feather, bones, and feet (Prakash et al. 2023). Cemani Chickens breeding begins with the process of incubating the eggs for 21 days. The process of breeding Cemani Chickens is divided into three phases, namely the starter phase where the chickens are 1 ± 40 days old and the second is the grower phase where the chickens are two to six months old. At this age, Cemani Chickens have begun to be selected for ornamental chicken purposes and as preparation for replacement of rejected broodstock. In this phase the chickens are separated according to feather type and tongue color for selection and selling price purposes. There is also a final phase, namely the layer phase where the chickens will be separated for reproductive purposes and abandoned (Habsari et al. 2019). This shows that the Cemani Chickens breeding process requires more attention compared to other chicken. Genetically, Cemani Chickens also have a longer growth period (Prakash et al. 2023).

In terms of feed, Cemani Chickens also receive more attention. Cemani Chickens aged 1–3 days are only given drinking water with added palm sugar. The purpose of giving brown sugar is to increase the nutritional source or energy source for DOC through drinking water so that it is easily absorbed and the chicken's stamina can increase (Aryanti et al. 2013). At the age of 3–40 days, Cemani Chickens are given feed with a protein content of 22% and energy of 3050 kcal/kg. The layer period in Cemani Chickens is from 6.5 months of age until they are laid off, given ground corn, rice bran and egg concentrate in a ratio of 3:4:3 feed and given additional minerals of 0.2 g in 10 kg with a protein content of 11.64%. and EM 3598 (Rofii et al. 2018). The feed composition and nutritional content in the grower and layer periods are different because the protein needed is used for different needs. Providing 17% protein at the age of 14–20 weeks or before the egg-laying period can have a positive impact during the reproductive period (layer). However, if the ration protein level is too low it will also cause slow growth. On the other hand, if the diet protein level is too high then growth will increase, but not commensurate with the costs of increasing diet protein (Habsari et al. 2019). The detailed nutrition content of feeding Cemani Chicken ensures that the nutritional content of Cemani Chicken is higher. Cemani Chickens are also believed to be trustworthy as sacrificial animals in traditional and religious rituals and are believed to be beneficial for health (Nguyen et al. 2023). This is because the black color of the Cemani Chicken indicates the high melanin content in the Cemani Chicken's body which has an impact on the high protein and fat content, as well as being rich in antioxidants and glucose-binding properties (Nguyen et al. 2023). The many benefits and uses of Cemani Chicken have caused consumer interest in Cemani Chicken to be very high. Apart from that, the complexity of Cemani Chicken breeding management, feeding and disease control also causes the price of Cemani Chicken meat and eggs to be very high compared to other types of chicken (Prakash et al. 2023). Table 7 provides an overview of the price of the different species of chicken.

**Table 7:** The price and feed of different chicken species around the world

Chicken species	Price	Feed price	Reference
Indonesia			
Broiler chicken	Rp35.350/1 chicken	Rp11.500/kg	https://www.bi.go.id/hargapangan and https://www.tokopedia.com/easy4store/pakan-popan-ayam-511-repack-1kg?extParam=ivf%3Dfalse&src=topads
Free-range chicken (ayam kampung)	Rp55.600/1 chicken	Rp7,000/kg	https://jepin.pontianak.go.id/harga_komoditas/daging-ayam-kampung and https://www.tokopedia.com/milenial-farm/pakan-ayam-pedaging-buras-pakan-ayam-kampung-b12l-1000gram-500-gram?extParam=ivf%3Dfalse%26src%3Dsearch
Sentul Chicken	Rp28.000/kg	Rp12.000/kg	https://urangciamis.blogspot.com/2012/12/ayam-sentul-asli-ciamis-unggas-lokal.html and https://shopee.co.id/PUR-AYAM-BRAVO-511-PUR-AYAM-KILOAN-PUR-AYAM-PEDAGING-i.694808684.12595746218?sp_atk=646c1df7-c128-47d0-bc66-806406298b46&xptdk=646c1df7-c128-47d0-bc66-806406298b46
Pelung Chicken	Rp1.183.000/1 chicken	Rp11.900/kg	https://www.blibli.com/p/paket-sepasang-ayam-pelung-jumbo-siap-produksi/ps--HAS-70760-28242 and https://jualayamhias.com/makanan-ayam-pelung/
Cemani Chicken	Rp35.000.000/1 chicken	Rp155.000/kg	https://jualayamhias.com/jual-ayam-cemani-dari-bibit-sampai-dewasa-siap-di-ternakan/ and https://shopee.co.id/NANJUNG-105-VOER-PUR-PELET-EBOD-100GRAM-PAKAN-BURUNG-KACER-MURAI-i.694808684.13995700782?sp_atk=3a16532b-81ea-4617-808e-a3a2f0f416ae&xptdk=3a16532b-81ea-4617-808e-a3a2f0f416ae
Siam Chicken	Rp170.000–350.000/chicken	-	Ayam Siam di Banda Raya—OLX Murah Dengan Harga Terbaik—OLX.co.id
Black Kedu Chicken	Rp850.000/pair	-	Mengenal Ayam Kedu, Kelebihan hingga Bedanya dengan Cemani (putraperkasa.co.id)
Bekisar Chicken	Rp 959.900/ 1 chicken	Rp 12.000/kg	https://shopee.co.id/Induk-Ayam-Jago-Bekisar-Indukan-Ayam-Hias-Turunan-Ayam-Hutan-Hijau-Dewasa-Jantan-Bandung-i.849673200.18839483256?sp_atk=f08d4fb2-c1b0-4aa4-9705-5ef30cbf72d2&xptdk=f08d4fb2-c1b0-4aa4-9705-5ef30cbf72d2
Vietnam			
Dong Tao Chicken	Rp17.500.000/1 chicken or $2500 per pair	-	https://www.chickensandmore.com/11-most-expensive-chicken-breeds-in-the-world/#2_Dong_Tao
India			
Kadaknath Chicken	Rp17.500.000/1 chicken or $2500 per pair	-	https://www.chickensandmore.com/11-most-expensive-chicken-breeds-in-the-world/#5_Kadaknath
Sweden			
Swedish Black Chicken	Rp4.200.000/1 chicken or $100	-	https://natureofhome.com/most-expensive-chicken-breeds/#:~:text=Swedish%20Black%20chickens%20have%20adapted%20well%20to%20the,to%20grow%20higher%20and%20higher.%20Chicks%20cost%20%24100
Olandsk Dwarf Chicken	Rp1.500.000/1 chicken or $100 +	-	https://www.chickensandmore.com/11-most-expensive-chicken-breeds-in-the-world/#9_Olandsk_Dwarf
France			
Breese Chicken	Rp2.800.000/1 chicken $10 per chick	-	https://www.chickensandmore.com/11-most-expensive-chicken-breeds-in-the-world/#7_Bresse
Belgium			
Liege Fighter Chicken	Rp2.100.000/1 chicken $55-$100	-	https://www.chickensandmore.com/11-most-expensive-chicken-breeds-in-the-world/#3_Liege_Fighter
United States			
Brahma Chicken	Rp2.100.000/1 chicken $7 each	-	https://www.chickensandmore.com/11-most-expensive-chicken-breeds-in-the-world/#11_Brahma
Turkey	Rp 350.000/ 1 chicken	Rp 12.000/kg	https://mocipay.com/8324/harga-ayam-kalkun-anakan-dan-dewasa/
Malaysia			
Serama Chicken	Rp500.000–25.000.000/1 chicken	-	https://www.roysfarm.com/serama-chicken/#:~:text=How%20much%20does%20a%20serama%20cost%3F%20Exact%20price,even%20exceed%20%24100%20each%2C%20depending%20on%20their%20broodline
Netherlands			
Poland Chicken	Rp4.000.000/1 chicken or $4–5 per chick	-	https://www.chickensandmore.com/polish-chicken/#Summary
China			
Silkie Chicken	Rp3.500.000/1 chicken	Rp 12.000/kg	https://ternakhias.com/content/detail/94/ayam/ayam-american-silkie
UK			
Orpington Chicken	Rp 4.000.000/ pair of chicken	Rp 12.540/kg	https://ternakhias.com/content/detail/17/ayam/ayam-orpington
Cochin Chicken	Rp. 1000.000/ 1 chickens	Rp 12.000/kg	https://www.putraperkasa.co.id/blog/apa-itu-ayam-cochin-asal-ciri-jenis-harga/
Italy			
Leghorn Chicken	$ 15- $30 / 1 chicken	Rp 52.500	How Much Do Leghorn Chickens Cost? (birdvenue.com)
Germany			
Phoenix Chicken	Rp. 1.335.000/pairs	Rp 10.300/Kg link	https://www.ternakhias.com/content/detail/12/Ayam/ayam-phoenix

## Assessment of Ayam Cemani environmental impact by life cycle assessment

**Fig. 7:**
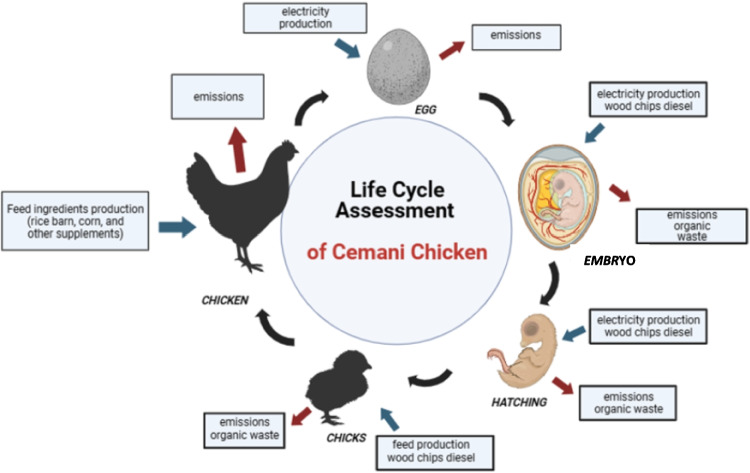
LCA stage of cemani chicken

There is an urgent requirement to assess the environmental implications of various food production systems in the current context of constantly increasing food consumption (Notarnicola et al. 2015). There are several approaches to manage the environment impact, including Life Cycle Assessment (LCA), eco-efficiency, and clean output (Azmi et al. 2023). In order to increase efficiency and minimize environmental harm, cleaner manufacturing incorporates environmental management into products, services, and processes. Eco-efficiency is a strategy to maximize resources while improving their environmental impact. A LCA can be used to determine a product's potential lifetime impact on the environment in the meantime (Azmi et al. 2023). LCA's primary advantage is that it allows to examine a product's whole life cycle and all of its consequences on the environment by taking consideration the entire life cycle of the pertinent product or system (Notarnicola et al. 2015) (Fig. 7).

The life cycle method has four stages: objective and scope setting, life cycle inventory analysis, life cycle impact assessment, and life cycle interpretation. It is founded on the ISO 14040:2006 LCA concepts and framework. To determine the greenhouse gas (GHG) emissions from broiler meat, LCA is commonly used (Mostert et al. 2022). A type III eco-label or Environmental Product Declaration (EPD) can be made using the LCA calculation findings. Eco-labels will increase a product's competitiveness in domestic and international markets (Azmi et al. 2023).

The LCA method has been used in a number of studies to evaluate the environmental effects of the poultry industry. Most of these investigations are still confined to a single boundary system or the cradle-to-gate system boundaries. For instance, Mostert et al. (2022) solely assessed the environmental effect inside the confines of the chicken farm. While, Kalhor et al. (2016) and González-García et al. (2014) looked into the effects of the boundaries between slaughterhouses and chicken farms. In addition, several studies have looked at the situation from the cradle to the slaughterhouse gate, and even more research have looked at the situation from the cradle to the grave borders (Azmi et al. 2023).

Rearing Cemani Chickens is basically the same as rearing free-range chickens (Ayam Kampung) as shown on Fig. 7. In general, Cemani Chicken breeders hatch Cemani’s eggs naturally. According to NF Farms, Cemani Chicken eggs can be hatched using an incubator, but their hatchability is low, only around 50–60%, but if they are hatched naturally, the hatchability can reach 70–80% (Habsari et al. 2019). According to Rofii et al. (2018), the difficulty of knowing the broodstock’s incubation temperature because Cemani Chickens differ from free-range chickens in specific ways, which leads to temperature regulation in the hatching machine.

**Fig. 8:**
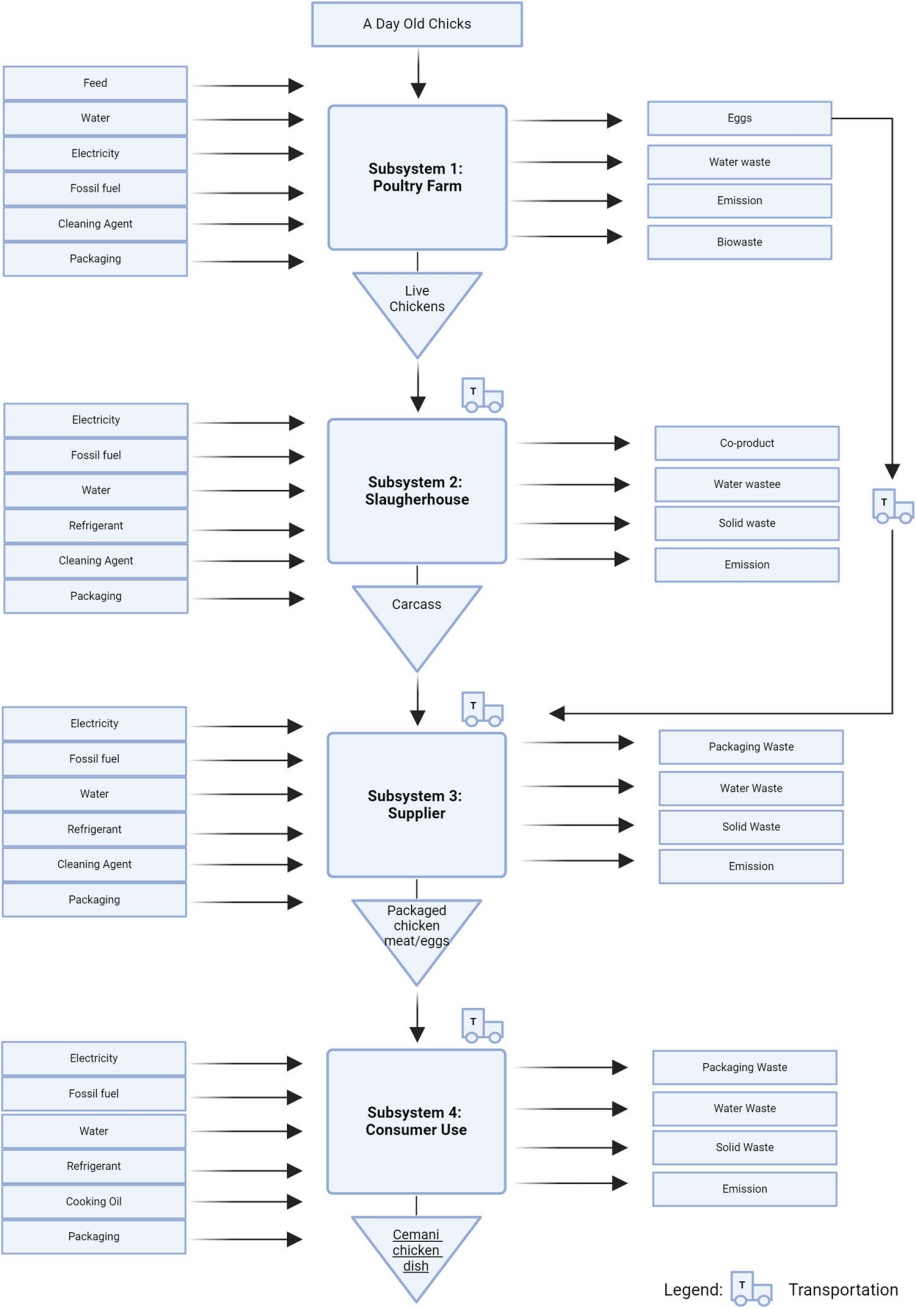
System boundary of cemani chicken meat production

But the Cemani Chicken rearing process not significantly different from other species of chickens. The consumption of energy and resources in the chicken meat production chain results in waste and greenhouse gas (GHG) emissions as shown on Fig. 8 (Azmi et al. 2023).

Global warming potential (GWP), acidification potential (AP), eutrophication potential (EP), ozone layer depletion (OLD), photochemical smog (PS) and human toxicity (HT), abiotic depletion potential (ADP), land competition use (LC), formation of photochemical oxidants (POFP), energy consumption (EC), water consumption (WC), cumulative non-renewable fossil and nuclear energy demand (CED), terrestrial ecotoxicity (TEP), freshwater depletion (FD), and freshwater aquatic ecotoxicity (FEP) are some of the categories used to categorize the environmental impacts (Azmi et al. 2023). According to Skunca et al. (2015), the common potentials assessed for the slaughterhouse, meat processing plant, retail, and household use subsystems are GWP, AP, EP, and OLD; for the poultry farm subsystem, GWP, AP, and EP; and for the slaughterhouse subsystem, is GWP. Having one kilogram of fried chicken resulted in 5.86 kg CO2 equivalent of global warming, 38.3 g SO2 equivalent of acidification, and 24.1 g PO43-eq of eutrophication. The production of feed, litter, and energy usage were the main causes of environmental problems. Lowering crude protein in feed, composting litter, installing inverters on refrigeration compressors, and boosting electrical energy efficiency were among options for reducing their negative effects on the environment (Azmi et al. 2023).

There are at least four subsystem boundaries in poultry meat chain production, including Cemani Chickens. The major subsystem boundaries of poultry meat chains are poultry farms. In Indonesia and others Asian countries, chickens mostly are bred in a traditional/backyard type. Backyard chicken farming is regarded as a traditional system in Indonesia due to its short production cycle and minimal investment requirements (Lan Phuong et al. 2015). In particular, there are no papers that look at how the Cemani Chickens meat chain affects the environment. But Lan Phuong et al. (2015) used LCA on an integrated agriculture–aquaculture (IAA) farming system in Indonesia with rice, fruit trees, veggies, pigs, chicken, and fish. The data showed that the impact per kg of pig and poultry protein was on average 1.6–1.8 times higher than the impact per kg of fish protein in each impact category (GWP, EP, and AP). The environmental effects of raising industrial broiler chickens and farmed salmonids (salmon, marine trout, and Arctic char) can be lessen the impact on the environment by raising broiler chickens uses up 9 times as much land as raising fish (~ 924,000 km^2^ vs. ~ 103,500 km^2^), however it cost 55 times money than regular rearing method (Halpern et al. 2022).

Poultry farming had the highest value-added, lowest eco-efficiency, and largest water impact (Giusti et al. 2022). González-García et al. (2014) found that the chicken farm caused the most environmental damage. Feed production and on-farm emission were the main environmental hotspots. Global warming potential (GWP), acidification potential (AP), and eutrophication potential (EP) are common potentials for poultry farm subsystems (Skunca et al. 2015). Feed production is the major environmental impact of chicken farms. GWP, CED, AP, and EP show the farm's largest environmental impact. The slaughterhouse, retail, and household directly impact energy consumption, while the meat processing facility largely impacts packaging materials and energy use.

The environmental impacts produced in the slaughterhouse subsystem were from energy (electricity and diesel), packaging materials, cleaning materials, refrigerants, and production waste (wastewater, biowaste, and packaging waste). Electrical energy was the most major contributor to global warming (0.94 kg CO_2_ eq (87.48%)), acidification (4.07 g SO_2_ eq (87.34%)), and eutrophication (4.98 g PO_4_^3−^ eq (82.96%)) in slaughterhouse subsystem (Azmi et al. 2023). Previously, Kalhor et al. (2016) evaluated the environmental impact of broiler production at farm gate and chicken meat production at slaughterhouse gate per mass-based functional unit in summer and winter seasons in Varamin city of Tehran province, Iran, using LCA method. The environmental impact for production of 1 ton packed meat in summer was estimated to contribute to GWP as much as 2931.91 kg CO^2−^eq, to AP as much as 41.75 kg SO^2−^eq, and to EP as much as 14.69 kg PO^4−^eq, meanwhile in winter was 5357.61 kg CO^2−^eq, 61.9 kg SO^2−^eq and 19.34 kg PO^4−^eq, respectively (Kalhor et al. 2016).

Furthermore, in the supplier subsystem, energy (electricity and diesel), packaging materials, refrigerants, and waste (wastewater and packaging waste) were the source of emissions. Environmental impacts were generated in the consumer use subsystem due to energy (electricity, LPG, and diesel), cooking oil, refrigerant, and waste (solid waste and wastewater). The use of electricity was the major contributor to the environmental impact of global warming (0.10 kg CO_2_ eq (45.66%)), acidification (0.29 g SO_2_ eq (48.01%)), and eutrophication (0.65 g PO_4_^3−^ eq (56.84%) (Azmi et al. 2023).

Besides that, feed production in chicken farming is very climate intensive since feed ingredient manufacturing has the greatest negative environmental impact (Giusti et al. 2022; Mostert et al. 2022; Azmi et al. 2023). The environmental impacts in the feed production subsystem were caused by the manufacture of feed ingredients (maize grain, soybean meal, wheat bran, rice bran, fishmeal) and energy use (electricity and natural gas). Soybean meal production contributed the most to global warming (1.17 kg CO_2_ eq (39.93%)). In this subsystem, however, maize grain production was the most important contributor to acidification (7.24 g SO2 eq (39.61%)) and eutrophication (3.52 g PO43- eq (42.56%)) (Azmi et al. 2023). Not only in poultry farms, but feed production also has the greatest environmental impact in pig farms. The feed for the pigs is responsible for a major part of the environmental impacts. More specifically, maize and soya are the components with the highest environmental impacts due to factors such as transportation, use of fertilizers and diesel fuel (Lamnatou et al. 2022).

However, the used of commercial feed can be replaced with insect such as black soldier fly (BSF) larvae. According to Heines et al. (2022) tried replacing some commercial feed with live black soldier fly larvae (BSFL) to reduce feed production's environmental impact. The life cycle assessment showed that supplemented BSFL in the feed increased yields in both scenarios (laying hen: 11.2 mPt per kg protein; broiler chicken: 9.63 mPt per kg protein), with fruit and vegetable waste-fed BSFL performing slightly, but not significantly, better (laying hen: 11.16 mPt per kg protein; broiler chicken: 9.57).

Reducing feed use per kilogram of broiler produced directly reduces the impact on the environment. The performance of conventional buildings and tunnel-ventilated houses differed significantly. The weight gain and higher feed conversion ratio of birds housed in tunnel-ventilated housing were observed. Additionally, the quantity of feed consumed influences the amount of manure produced, which in turn influences emissions from homes and fields (Kalhor et al. 2016) (Fig. 8).

## Comparison of nutritional value between Ayam Cemani and other main chicken breeds present in Indonesia

Chicken meat’s consumption in Indonesia has the highest rank compared to beef and lamb (Licorice 2020). Due to its low fat, high protein, and minimal religious and cultural restrictions, make chicken meat dominates choice meat eating by Indonesians. People’s awareness of the healthy lifestyle and the demand of low-cost protein food, make the chicken products such as meat and eggs increase since meat and egg have complex and balanced nutrition for human health (Afrianti et al. 2013; Mueller et al. 2018; Kim et al. 2020). In Indonesia there are several popular chickens which can be found and consumed. The nutritional value of popular chicken in Indonesia is shown in Table 8.

**Table 8:** Nutritional profile of five Indonesian main chicken species

No	Chicken breed	Muscle mass (%)	Protein content (%)	Lipid content (%)	Total mineral (%)	Reference
1	Broiler chicken (meat)	Not found	17.4	16	0.8	(Mir et al. 2017)
	Broiler chicken (egg)	-	13	12	0.8	(Nova et al. 2014)
2	Native Chicken (ayam kampung)	Not found	17.1	2.43	4.5	(Soriano-Santos 2010)
	Native Chicken (egg)		16.3	11.5	4.0	(Halendra et al. 2011)
3	Pelung Chicken	Not found	Not found	Not found	Not found	-
4	Sentul Chicken	Not found	15%	5.3	Not found	(Masito et al. 2019)
5	Cemani Chicken	45–47	18	3	13.5	(Łukasiewicz et al. 2015; Kostaman et al. 2021)

One of the crucial elements required to promote the development of chicken body tissue is feed. Good quality feed is necessary to provide the nutritional needs for improved growth and to boost endurance in order not only to promote the productivity of maintained chickens but also to enhance the quality of their eggs and meat (Marla et al. 2021). Cemani Chicken has a high muscle mass. It occurs when the feed of Ayam Cemani is controlled. Muscle growth is affected by intrinsic factors (e.g., genetic factors) and extrinsic factors such as nutrients, metabolism, sex, hormones, and activity. Energy, protein, fat, crude fibres, vitamins, minerals, and amino acids must all be present in suitable amounts in the feed that hens are fed, (Deviana et al. 2018). Maximum growth patterns can only be supported by effective feeding and sufficient nutrients (Hanafi et al. 2021). The amount of muscle mass in chicken meat is similarly influenced by feeding frequency. Chickens are typically fed twice a day, from 6–7 am until 1–2 pm (Nguyen et al. 2023). Thus, the quantity of muscle mass in chicken meat is also influenced by body weight. For instance, chicken with slower-growing genotypes had fewer breast and thigh muscle yields but had greater protein contents (Mikulski et al. 2011). Additionally, free-range chickens are more active outside, they have higher protein levels but are also less juicy and have a darker color than chickens bred indoors (Caldas et al. 2019). Moreover, even there’s no exact number of native chicken’s muscle mass, native chicken known has smaller body appearance compared with broiler chicken and Cemani Chicken. This because native chicken generally lives freely and they search their own food, differ with broiler and Cemani Chicken (Nguyen et al. 2023).

The amount of protein content in chicken meat is influenced by the protein content contained in the feed given. Feed that contains high protein will increase the protein content in meat. According to Le et al. (2020), the protein contained in laying hen feed is 142.50 g/kg and the protein contained in broiler feed is 19–22.5%. Broilers can take 20–25% of their protein needs from the environment (Le et al. 2020).

However Cemani Chicken has a lower lipid content (3%) compared with broiler chicken (16%), but higher when compared with Ayam Kampung (2.43%). The lipid content found in the chest muscle of male Cemani Chicken ranges from 0.17–0.21%, while in broiler and native chicken ranges from 1.07–8.1% (Hidayat and Asmarasari 2015). The amount of lipid content in chicken meat is also influenced by the gene that is expressed. In poultry, FTO-regulated gluconeogenesis in DF-1 cells seems to be responsible (Guo et al. 2015). FTO mRNA expression was identified in muscle tissue, liver, visceral fat, hypothalamus, cerebellum, and telencephalon, and there were significant differences in liver tissue and visceral fat in broilers and layers (Wang et al. 2012). The lower fat content in Cemani Chicken makes it popular among consumers who want to eat low-fat chicken. Chicken activity can also affect the lipid content of chicken meat. Chicken activities such as scavenging, dust bathing, and perching will form muscle tissue in chickens. The higher the muscle tissue that is formed, the higher the chicken carcass weight will be. Sadly there is no information about carcass weight of Cemani Chicken available (Wang et al. 2012).

The total minerals contained in the breast muscle of the Cemani Chicken is greater than that of the broiler chicken and native chicken. Based on the research results of Kostaman et al. (2021) the total minerals found in the breast muscle of the Cemani Chicken is 1.35%, while in broiler and native chicken are 0.8%-4.5%. Mineral data of the other chicken species has not been found. The high total minerals in chicken meat are influenced by the feed given to the chickens. Supplements given to chickens can also affect the total minerals in chicken meat. The most abundance mineral on Cemani Chicken are potassium (420.0 mg), calcium (6.0 mg), and sodium (215.0/mg) (Kostaman et al. 2021).

In the maintenance of broiler and livestock, liquid supplements are given which contain minerals that are important for the growth of bones, external and internal organs, blood formation and others. The main amino acids, such as arginine, histidine, isoleucine, lysine, methionine, tryptophan, and valine as building blocks of protein for the formation of cells, tissues and organs. As well as complete vitamins, namely A, D, E, K, C and B complex for health and body resistance. Generally, supplements are given by adding feed ingredients and drinking water to livestock (Hudák et al. 2021).

Protein is the main nutrient for chicken meat and egg. Protein has various important functions in the body, including growth, development and maintenance of almost entirely body parts such as skin, tendons, membranes, muscles, organs and bones as well, supports repair of body tissue and acts as body antibodies (Ariany et al. 2012). Cemani Chicken has higher protein content (18%), compared with broiler chicken (17.4%), Ayam Kampung (17.1%), Sentul Chicken (15%). In addition, based on the research from Kostaman et al. (2021), Monounsaturated Fatty Acid (MUFA) and linoleic acid on egg yolk of Cemani Chicken is higher than the egg yolk of white leghorn chicken species, which is 39.66% and 14.54%, while on white leghorn only 36,16% and 13.97%. (Magara et al. 2021). Compared the other chicken species, Cemani Chicken have the highest protein and mineral content. The high protein and mineral content shown that Cemani Chicken is healthier for consumption than the other species (Kostaman et al. 2021).

Beside the comparison of nutritional value between Cemani Chicken and the other chicken species in Indonesia that already mentioned above, Cemani Chicken known have another advantage that came from its black color. The black color of Cemani Chicken caused by melanin content that accumulated on Cemani Chicken’s body (Nguyen et al. 2023). The primary phenolic compounds that give black food its color are called melanin. The biological pigment known as melanin is frequently seen in the skin, hair, and eyes. Numerous biological systems, such as those of plants, insects, seafood, and even bird feathers, contain it (Nguyen et al. 2023). Potential sources of phytochemicals that can significantly influence a number of key metabolic disorders, such as diabetes, obesity, cancer, osteoporosis, and menopausal diseases, are foods that are black in color (Leitzmann 2016). Additionally, a lot of anthocyanins in black food have strong antioxidant qualities and are associated with anti-inflammatory, vaso protective, antineoplastic, radiation-protective, chemo-, and hepatoprotective effects (Nganvongpanit et al. 2020; Nguyen et al. 2023). Black foods often contain higher amounts of antioxidant activity than other colors within the same species in certain situations. This has implications for the food, pharmaceutical, and cosmetic industries. When it comes to eggplants, black and purple cultivars are said to have higher total phenolic contents and antioxidant qualities than white cultivars (Colak et al. 2022). Thus, Cemani Chicken are recommended to be consumed as an alternative protein source than broiler chicken and the other chicken species in Indonesia.

## Methods to cook Ayam Cemani chicken meat

Cemani Chicken can be processed in several techniques in order to convert raw chicken into a consumable form and microbiologically safe (Choi et al. 2016). In general, food processing always utilizes hot temperatures. This can cause changes in the physio-chemical characteristics of chickens such as size, moisture content, ash content, protein content, fat content, and others (Sundari et al. 2015; Sari et al. 2016). The most popular techniques to cook Cemani Chicken are steaming and boiling. However, Cemani Chicken can be cooked and served in several techniques which can be seen in Table 9.

**Table 9:** Cooking methods for preparing Ayam Cemani meat

Cook/serve technique	Treatment	References
Boiling	Whole chickens body part were boiled in water for 4 h	(Solyakov and Skog 2002)
Stewing	A pot was assigned to a specific stewing time for each replication. A set of 10 chickens was stewed for each tested cooking time: 1, 2 or 3 h. The temperatures of the breast and thigh meat were between 30 to 35 °C at the start time, reached 93 to 95 °C after stewing for 25 min, and were kept within 93 to 98 °C for the rest of stewing	(Qi et al. 2018a)
Steaming	Samples were cooked at 100 °C under forced air circulation (air speed: 0.90 ± 0.13 m/s). Cooking treatments ended when meat samples reached 74 °C at their thermal centre, that is the recommended safe temperature for turkey meat (United States Department of Agriculture (USDA) & Food Safety & Inspection Service, 2006). Cooked samples were then cooled at room temperature for 30 min to reach a temperature of 27 ± 2 °C at their core	(Głuchowski et al. 2020)
Frying	Each portion was further subdivided into three different cooking temperature regimes (170, 180 and 190 °C) and each temperature portion further subdivided into five different frying time intervals (0, 4, 8, 12 and 16 min). Each of the samples was weighed and fried in four litres of canola oil, which previously preheated at 170 °C for 2 h for deep fat frying, and air fryer was preheated at 170 °C for 30 min	(Alugwu et al. 2022)
Baking	About 100 g raw chicken thigh meat was fully wrapped with the respective edible films (100 × 150 mm), Food Science and Technology International 0(0) 2 prepared with and without cumin and oregano oleo-resin. The control and wrapped samples were placed on plates and closed with stretch film. All the samples were stored at 4 °C for four days, followed by roasting at 200 °C for 30 min, in an air convection oven	(Küçüközet and Uslu 2018)
Grilling	Grilling took place on a singleside contact grill set to 200 ± 20 °C, with the slices of meat being turned when an internal temperature of 40 °C had been attained	(Ježek et al. 2019)
Pressure cooking	Pressure-cooking chicken breast was placed in a pressure cooker and cooked for 10 min (reaching 121 °C without holding time). After cooking and cooling, samples were vacuum-packed and stored at 20 °C until laboratory analysis was carried out	(Muthulakshmi et al. 2021)

Poultry products experience various changes during heat treatment such as weight loss, water holding capacity, texture, color and aroma development. In addition, the quality characteristics of the product are affected by the cooking method, time, and temperature (Choi et al. 2016).

First way of processing Cemani Chicken is boiling, water convection cooking techniques. Boiling require the meal for a brief length of time to be in boiling water. Boiling chicken can be a healthy choice since it doesn’t increase fat content. But it can reduce vitamin B, C and protein since those nutrient dissolve in water. According to Sundari et al. (2015), broiler chicken protein content is reduced by 1.65% when its boiled. Additionally, boiling can cause the protein in high protein meals to coagulate. While study by Javadi, (2011), shows that boiling can lower the residue levels of doxycycline and enrofloxacin antibiotics that has the potential to be dangerous to human if it consumed above the Maximum Residue Limits (MRLs).

Another technique to cook Cemani Chicken and poultry meat is stewing (Qi et al. 2018b). It refers to the shimmering of food in a pan with a tight fitting lid using small quantities of liquid to cover only half the food. This is a slow method of cooking. The liquid is brought to boiling point and the heat is reduced to maintain simmering temperatures (82^o^ C—90 °C). The food above the liquid is cooked by the steam generated within the pan. Since the water used for cooking is not discarded, nutrient loss using this method can be avoided and the flavour can be maintain. Stewing chicken meat can activate the volatile compound (Qi et al. 2020). Maximum chicken scent rating achieved after three hours of stewing. After stewing for two hours, the overall taste characteristics tend to stabilize. Generally speaking, stewing intensifies the scent of chicken flesh but weakens its flavour, especially in the first two hours (Qi et al. 2018b). However this method is time consuming and waste a lot of fuel.

Steaming is another method of cooking and serving chicken that uses water convection. In contrast to other forms of cooking, steaming solely uses the hot steam produced by water (Atmoko and Krestanto 2017). But up to this point, not a single article has described how Cemani Chicken is processed using this method. The references that were found are primarily concerned with how steaming affects processed chicken meat products like steaks and sausages (Asmaa et al. 2015; Choi et al. 2016). According to research findings Choi et al. (2016), steamed chicken steak has a higher water content than food prepared using other methods.

Processing chicken meat, is not only limited to water-based cooking techniques, but also oil or fat based. Frying is one of the method that brought the food into contact with large amount of hot fat or oil (Sundari et al. 2015). When food is totally immersed in hot oil, it is called deep fat frying, while in a shallow fat frying, only a little fat is used and the food is needed to be turned in order to make the both side cooked. However, there’s no research found that says Cemani Chicken can be fried but some people already cook Cemani Chicken with frying method. Frying method in Indonesia, are common to be used in broiler chicken and native chicken. The adverse effect on frying method is it can be less healthy since it increase fat and cholesterol content. Changes in the quality of fried chicken are significantly influenced by the frying temperature, duration, and oil to chicken thigh ratio. Lower peroxide values (PV) are the result of higher frying temperatures and longer frying periods. With increased chicken servings, P-AV rises noticeably (Sundari et al. 2015). This is inconsistent with reducing the ash content to 0.54%, protein content to 2.97%, and water content to 16.74%. To prevent raising levels of unhealthy cholesterol, avoid frying with repurposed oil (Sundari et al. 2015).

Dry heat technique such as baking and grilling can also be used to prepare the Cemani Chicken. Baking is a food preparation method that uses hot air vapor convection in an oven. Although it passes through the oven's apertures, the heat energy of the oven does not directly contact the food. The baking process usually utilizes a heating element/fire under the oven (Atmoko and Krestanto 2017). Increasing the roasting temperature of the meat can lower the value of cooking loss, organoleptic quality (color, smell, and taste), and texture value (measured using the concept of pressure or pull), according to the findings of research Radiati et al. (2017), on free-range chicken. On the other side, it can make chicken meat's pH level higher. This is caused by a drop in the acidic group, which raises the meat's isoelectric point and raises the pH at the same time. The length of baking time also has an impact on the chicken meat. The pH value, textural value, and organoleptic quality of chicken meat decrease with increasing baking time. In addition, the baking time also affects the chicken meat. The longer the baking time, the lower the pH value, textural value, and organoleptic quality of chicken meat. As well as causing the value of cooking loss to increase. In terms of color, the increase in temperature and increase in time have no significant effect. The main determinants of meat color are myoglobin concentration and chemical status. Many factors affect meat color including species, breed, muscle type, sex, and age (Radiati et al. 2017).

Another dry-heat cooking method is grilling. Grilling or broiling refers to the cooking of food by exposing it to direct heat. In this method food is placed above or in between a red hot surface. Cooking loss increases linearly with temperature. The loss increases as the cooking temperature rises. This is also connected to the fact that grilling requires a higher temperature than baking. Hence, the cooking loss when grilling is greater (Atmoko and Krestanto 2017). But grilling can enhances flavour, appearance, and taste of the product, also requires less time to cook.

Cemani Chicken is processed via pressure cooking, also makes use of water convection and high pressure. To cook with this technique, a special pot that is closed is needed, so that no steam or liquid can escape. As the air pressure inside the pot rises, the boiling point of water rises and can even surpass 100 ºC (Atmoko and Krestanto 2017). Tenderness, collagen, texture, taste, acceptability, meat color, ash content, and protein content all rise as a result of the high-pressure cooking method (Devi et al. 2017). According to studies conducted by Devi et al. (2017) and Głuchowski et al. (2020), pressure-cooked chicken samples had a higher protein content than raw chicken samples. This is brought on by the greater moisture loss during cooking. However, according to studies by Muthulakshmi et al. (2021) pressure cooking actually reduces the amount of water and fat in food. Levels of saturated fatty acids (SFA) decreased by 32.25%, those of monounsaturated fatty acids (MUFA) by 40.66%, and those of ratio polyunsaturated fatty acids (PUFA) n-6/n-3 increased by 17.89%. In terms of meat color, the difference in pressure used causes the resulting color to also be different. The color of the meat generated during cooking lightens with increasing pressure. This is so because sarcoplasmic and myofibril proteins, which have a big impact on the color of the flesh, can become denatured under high pressure (Marušić Radovčić et al. 2020). Other investigations' findings support the notion that flesh can bleach under pressures of more than 200 MPa. According to Simonin et al. (2012), protein coagulation and globin denaturation cause this whitening effect.

However, even the methods used to process Cemani Chicken come in various ways, the most common method used to process Cemani Chicken is boiling. Cemani Chicken meals are always processed with huge amount of traditional spices to enhance its flavour.

## Consumption and consumer’s acceptance of Ayam Cemani

Poultry is a source of protein that is required in order to improve the nutrition intake and support the body performance. Chicken is one of the most consumed protein source in Indonesia (Hidayat and Asmarasari 2015). Purebred meat chickens or broilers and native chickens (Ayam Kampung) are the two most popular types of chicken meat consumed in Indonesia (Hidayat and Asmarasari 2015). Indonesian people consumption of broiler chicken meat per capita/year in 2017 was 5.68 kg, an increase of 573 g (11.2%) compared to consumption in the previous year. Meanwhile, consumption of native chicken (Ayam Kampung) meat was 782 g per capita/year, an increase of 156 g (24.9%) from the previous year. The development of chicken-based culinary delights, from roadside stalls to shopping centers, has resulted in an increasing trend in chicken meat consumption throughout 2013–2017. In addition, broiler chicken meat production in 2017 reached 2.14 million tons, an increase of 97 thousand tons (4.75%) from the previous year which was only 2.04 million tons. Broiler meat production in 2013 only reached 1.54 million tons and continued to show an increase until 2017 (Magfira 2020).

According to a report by the Badan Pusat Statistik (BPS), in 2021 the average consumption of chicken meat in Indonesia reached 0.14 kg (kg) per capita per week (BPS 2022). In terms of trends, per capita consumption of chicken meat in Indonesia tends to increase during the 2011–2021 period. The highest growth rate was recorded in 2014, namely an increase of 19.76% from the previous year. The national average consumption of chicken meat is higher than beef or buffalo. It is recorded that the average consumption of beef or buffalo is only 0.009 kg per capita per week in 2021 (BPS 2022). According to a survey by Licorice (2020) on 500 Indonesians respondents aged 10–59 years old, chicken meat is the winner compared with the other meat. The high interest of Indonesian people in consuming chicken meat rather than beef or buffalo is due to a number of factors. Among other things, because the price is more affordable and production is more abundant. BPS noted that chicken meat production in Indonesia reached 3.42 million tons in 2021. Meanwhile, beef and buffalo meat production was 437.78 thousand tons and 20.97 thousand tons respectively last year (BPS 2022).

According to a report by BPS, throughout 2022 Indonesia will produce around 3.76 million tons of broiler chicken meat. In 2022, Central Java will become the largest broiler chicken meat producing province, while DKI Jakarta will be the only province with zero production. Based on BPS data, the average level of chicken meat consumption in Indonesia in 2022 will be around 0.15 kg per capita per week. This amount is higher than beef consumption, which averages only 0.01 kg per capita per week. However, the level of chicken meat consumption is lower than consumption of fresh fish and shrimp, which averages 0.37 kg per capita per week (BPS 2022; Adi Ahdiat 2023). Meanwhile, for native chicken (Ayam Kampung) meat production, East Java is the largest in Indonesia. This production reached 50,562 tons in 2020. Central Java was the second largest producer with production of 34,201 tons. West Java is in third position with production of 26,943 tons of free-range chicken meat (Pahlevi 2021).

Each type of chicken has its own unique characteristics and consumers. Broiler meat has a cheap selling price, starting from IDR 30,000/kg. Meanwhile, free-range chicken has prices starting from IDR 100,000/ kg (BPS 2022). From the texture of broiler chickens, it is easy to tenderize, unlike free-range chicken which requires time to tenderize. Another thing that differentiates native chickens (Ayam Kampung) from broiler chickens is their nutritional content (Nuswantoro 2020). Broiler chickens are richer in energy and fat because they contain more fat than native chickens (Ayam Kampung). Per 100 g of broiler chicken meat contains 295 kcal of energy and 14.7 g of fat. The size of free-range chickens tends to be smaller and thinner. Meanwhile, broiler chickens have fat and thick meat. This is because sometimes broiler chicken feed contains growth promotor that can boost its growth and body size (Marlina et al. 2015). In addition at the size of the breast, native chickens (Ayam Kampung) have protruding bones. Because the fat content of native chickens (Ayam Kampung) itself is less. Meanwhile, the breast part of a broiler chicken is fatter and denser. Almost no bones stick out. The choice is between consumer’s willingness to pay and taste. Because in terms of price, native chickens (Ayam Kampung) is more expensive. Meanwhile, broiler chickens are very affordable. However, if consumers look at it from a health perspective, native chickens (Ayam Kampung) is much more recommended because it has lower fat and grow naturally. Apart from its low fat content, many people say that native chickens (Ayam Kampung) meat is more delicious. In addition, consuming broiler chicken in a high amount can have adverse effect such as early puberty (Izza 2017).

On the other hand for Cemani Chicken, even though it is one of the varieties of native Indonesian chicken, there are not many archives about food made from Ayam Cemani. According to Sultan, Chairman of the Black Indonesian Community (BI), there is a restaurant in America that provides Cemani Chicken dishes at a price of IDR 35 million/head. Black Indonesia (BI) is the only community of Cemani Chicken fans and breeders in Indonesia (Antara 2018). Cemani Chicken has an expensive price because its meat has special properties. Cemani Chicken meat has a lot of benefit which is said to beneficial for health. Especially for Silkie Chickens, it is believed to strengthen the body's immunity. It is even said to be able to make a weak body stronger (Sharma et al. 2022). The black meat of Silkie chicken was found to contain twice as much carnosine concentrate (Sehrawat et al. 2021; Dharmayanthi et al. 2022). There are not many sources that explain the price of meat from Cemani chickens, but as reported by Webster, the price for a pair of adult Cemani chickens is IDR 535,000/pair. Ayam Cemani is a variety of free-range chicken. Not surprisingly, when cooked, black chicken meat does not fall apart easily but has a delicious taste. Cemani chicken meat has a light and savory taste with a slightly juicy texture, as quoted from the Tasting Table (Sharma et al. 2022) The black color of Cemani Chicken meat also makes the dish unique. However, the light taste of the meat means that Cemani Chicken can be cooked with any spices. Unfortunately, Cemani Chicken dishes are quite rare. Only a handful of chefs are interested in processing it.

From the explanation above, it can be concluded that Cemani Chicken meat is still relatively unpopular among Indonesian people and is still inferior to the popularity of broiler chickens and free-range chickens. This is due to the high price of Cemani Chicken and difficult access to obtain it because not many restaurants or eating houses provide Cemani Chicken meat on their menu. Apart from that, the price of Cemani Chickens is high because Cemani Chickens are considered to have beauty in their unique body shape, namely black from feathers, eyes to meat. So currently, Cemani Chickens are mostly reared as as ornamental chickens rather than as chickens for consumption. However, if Cemani Chicken meat is consumed, it has various benefits that are good for body health because this type of chicken meat contains high levels of selenium and minerals which are quite good in treating and overcoming arthritis. The mineral and vitamin content is also effective in improving the body's immune system (Sharma et al. 2022).

## Conclusions & future perspectives

Cemani Chicken is rare and has higher-quality meat than native or broiler chicken. This makes Cemani Chicken more expensive and in demand by both domestic and foreign customers. Cemani Chicken breeds grow slowly and produces less egg than native or broiler chickens. The good quality of the meat, the limited quantity on hand, and the strong demand from customers all together contribute to this chicken's expensive price. The price of Cemani Chickens might cost twice as much as other chickens in Indonesia. Cemani Chickens are only occasionally consumed because they are served during religious ceremonies as a sacrifice to the ancestors. To prove that the chicken's price is commensurate with its beneficial worth, however, scientific proof is needed. The raise of Cemani Chicken's competitiveness in both domestic and foreign markets can be increased by determining its nutritional content and quality.

In addition, the Cemani Chicken is still regarded as a unique breed and may only be raised in specific locations, even with its growing popularity. As a result, Cemani Chicken needs to be farmed and breed responsibly. Very little research has been done on Cemani Chickens so far. Studies that particularly address the genotype traits (combinations of alleles) of Cemani Chickens or other native Indonesian chickens are non-existent. Due to the dearth of information regarding the health of local chickens, it is challenging to assess how this affects the productivity and liveability of various breeds, including Cemani Chickens. Furthermore, no articles have particularly evaluated the environmental impact (life cycle assessment) of the production chain of Cemani Chickens meat, nor have they compared the environmental effects of various farming practices (conventional backyard, intensive, and semi-intensive). On the other hand, eco-labels will increase a product's competitiveness in domestic and international markets. To close the current knowledge gap regarding Cemani Chickens, more research has to be done.

## Data Availability

Not applicable.

## Abbreviations

LCA: Life cycle assessment
RJF: Red jungle fowl
*EDN3*: *Endothelin* 3 Gene
*BMP7*: Bone morphogenetic protein 7
PCR: Polymerase chain reaction
RFLP: Restriction fragment length polymorphism
AOBA: African ornamental breeders association
SFA: Saturated fatty acid
MUFA: Monounsaturated fatty acid
PUFA: Poly-unsaturated fatty acid
IDR: Indonesian rupiah
AGP: Antibiotic growth promotor
GHG: Greenhouse gas
EPD: Environmental product declaration
GWP: Global warming potential
AP: Acidification potential
EP: Eutrophication potential
OLD: Ozone layer depletion
PS: Photochemical smog
HT: Human toxicity
ADP: Abiotic depletion potential
LC: Land competition use
POFP: Photochemical oxidants
EC: Energy consumption
WC: Water consumption
CED: Cumulative non-renewable fossil and nuclear energy demand
TEP: Terrestrial ecotoxicity
FD: Freshwater depletion
FEP: Freshwater aquatic depletion
IAA: Integrated agriculture–aquaculture
BSF: Black soldier fly
BSFL: Black soldier fly larvae
FTO: Fat mass and obesity
RNA: Ribonucleic acid
